# Supplementary material for: ISRIB Blunts the Integrated Stress Response by Allosterically Antagonising the Inhibitory Effect of Phosphorylated eIF2 on eIF2B
Source: Mol Cell. 2021 Jan 7;81(1):88–103.e6. doi: 10.1016/j.molcel.2020.10.031 (PMC7837216; doi:10.1016/j.molcel.2020.10.031)
Supplement: Document S1. Figures S1–S4 and Tables S1–S3 [file mmc1.pdf]

Molecular Cell, Volume *81*

Supplemental Information

ISRIB Blunts the Integrated Stress Response by Allosterically Antagonising the Inhibitory Effect of Phosphorylated eIF2 on eIF2B

Alisa F. Zyryanova, Kazuhiro Kashiwagi, Claudia Rato, Heather P. Harding, Ana Crespillo-Casado, Luke A. Perera, Ayako Sakamoto, Madoka Nishimoto, Mayumi Yonemochi, Mikako Shirouzu, Takuhiro Ito, and David Ron

Supplemental materials

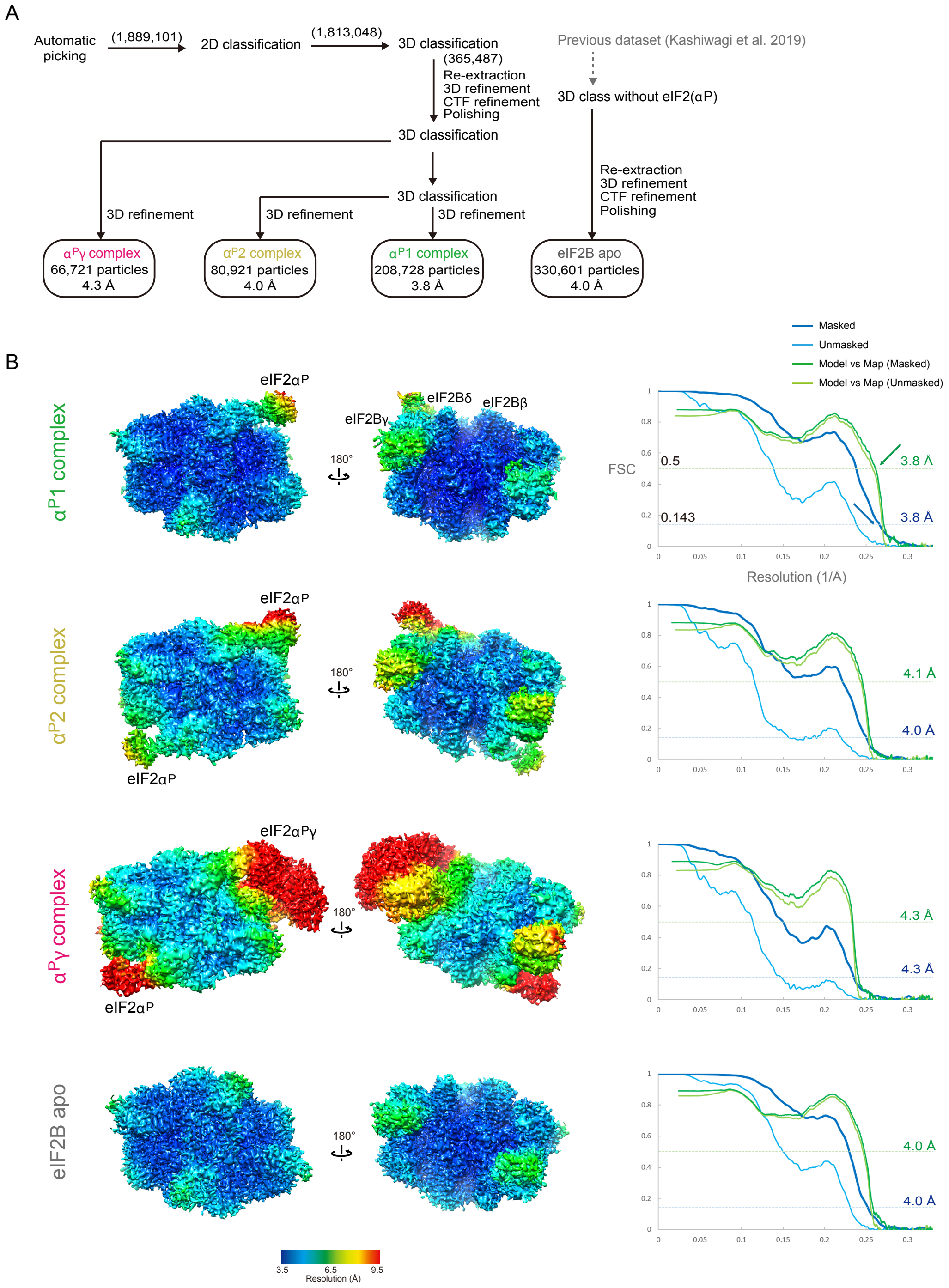

Figure S1. Cryo-EM data processing, related to Figure 2 and Table 1.

A) Workflow of image processing. Total particle numbers at each stage are show in parentheses.

B) Local resolution maps and Fourier shell correlation (FSC) curves of the cryo-EM maps. The FSC curves for masked (blue), unmasked (cyan) map, and the curves for model and map correlation (masked: green, unmasked: yellow green) are shown. The resolutions at which FSC for masked map drops below 0.143 and model map correlation drops below 0.5 are shown.

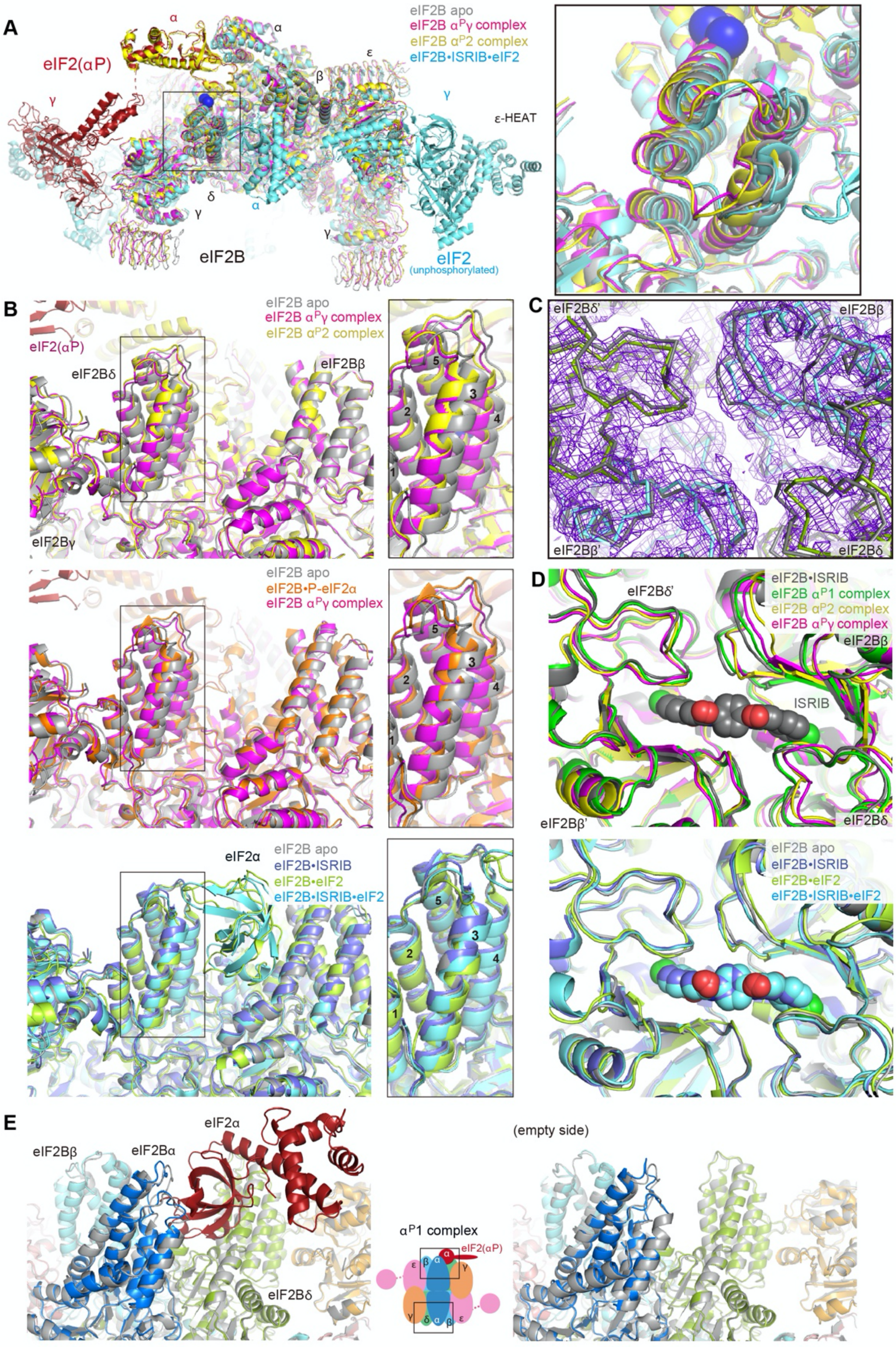

Figure S2. Changes to the catalytically productive interface and the ISRIB-binding pocket of eIF2B induced by eIF2(αP) binding, related to Figure 2.

A) Overlay of the eIF2B apo structure (grey), the $\alpha^P\gamma$ complex (eIF2B in magenta and eIF2(αP) in red), the α^P2 complex (all in yellow), and the eIF2B•ISRIB•eIF2 complex (all in cyan, PDB: 6O81) (same view as Figure 2A). Right panel: Close-up view of the displacement of the N-terminal helices of eIF2Bδ by the binding of unphosphorylated (cyan) or phosphorylated eIF2 (magenta). The blue spheres show the position of the C^α atoms of eIF2Bδ^{E310} and δ^{L314}.

B) Comparisons of the catalytically-productive pocket of eIF2B in various structures (same view as Figure 2B). Upper panels: comparison of apo eIF2B (grey) and eIF2B complexes with the eIF2(αP) trimer in which eIF2βγ is resolved ($α^Pγ$, magenta), or unresolved ($α^P2$, yellow). Middle panels: comparison of apo eIF2B (grey), the $α^Pγ$ complex (magenta) and the complex with the isolated phosphorylated eIF2α (P-eIF2α) (PDB: 6O9Z, orange). Lower panels: comparison of apo eF2B (grey), the eIF2B•ISRIB complex (PDB: 6CAJ, blue), the eIF2B•eIF2 complex (PDB: 6K71, lime), and the eIF2B•ISRIB•eIF2 complex (cyan). Right panels are close-up views of the helix δ-α3. Note that eIF2Bδ of the $α^P2$ complex shows a similar displacement of eIF2Bδ as the $α^Pγ$ complex structure (upper panels), while eIF2Bδ of the eIF2B•P-eIF2α complex resides in a position intermediate between apo eIF2B and the $α^Pγ$ complex (middle panels). In both the eIF2B•eIF2 complex and the eIF2B•ISRIB•eIF2 complex, eIF2Bδ closes around eIF2α, while there is little displacement of eIF2Bδ in the eIF2B•ISRIB complex (lower panels).

C) Close-up view of the ISRIB-binding pocket at the eIF2B's β-δ interface (same view as Figure 2C). The EM density map for the $α^Pγ$ complex is shown in purple.

D) Views of the ISRIB-binding pocket (same view as Figure 2C). Upper panel: the comparison of apo eIF2B (grey), the $α^P1$ complex (green), the $α^P2$ complex (yellow), and the $α^Pγ$ complex (magenta). Lower panel: comparison of apo eIF2B (grey), the eIF2B•ISRIB complex (blue), the eIF2B•eIF2 complex (lime), and the eIF2B•ISRIB•eIF2 complex (cyan). Note the similar displacement between the β-δ heterodimeric units in the $α^Pγ$ complex and the $α^P2$ complex, but not in other structures.

E) Comparison of the eIF2B$α_2$ homodimeric unit of the $α^P1$ complex (color-coded as in the adjacent cartoon) relative to the eIF2B apo structure (grey). Accommodation of a single molecule of eIF2(αP) induces only minor displacement at tips of the eIF2B$α_2$ homodimer.

Structures are aligned by the four C-terminal domains of the β- and δ-subunits of eIF2B for A), B), E), and by the $C^α$ atoms surrounding (within 10 Å) the ISRIB molecule in the eIF2B•ISRIB structure for C), D).

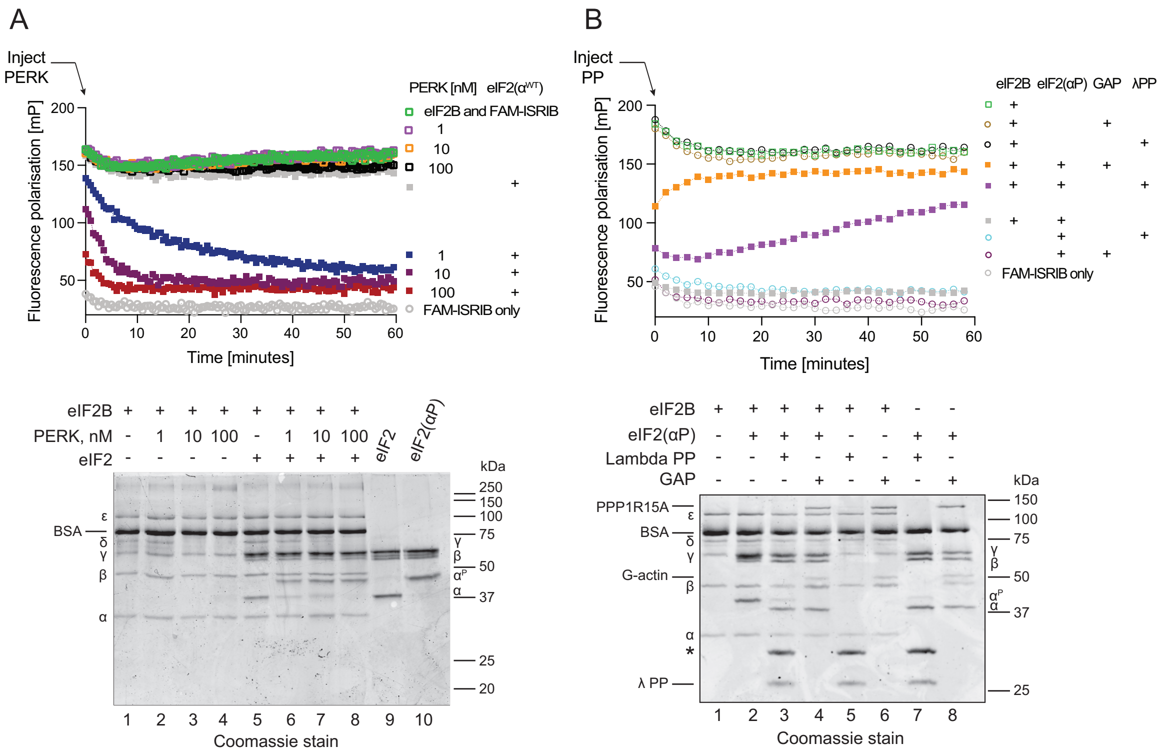

Figure S3. eIF2(αP)-mediated inhibition of FAM-ISRIB binding to eIF2B is captured kinetically and is reversible by dephosphorylation, related to Figure 4.

A) Upper left panel: plot of time-dependent change in fluorescence polarisation of FAM-ISRIB bound to wildtype eIF2B in presence or absence of unphosphorylated eIF2. Where indicated, at t = 0 the eIF2α kinase PERK was introduced at varying concentrations to promote a pool of eIF2(αP). Shown is a representative experiment (one of three).

Lower left panel: Coomassie-stained PhosTag SDS-PAGE of the samples analysed in the experiment above. Migration of the eIF2 subunits, including phosphorylated and unphosphorylated eIF2α, are indicated on the right. Pure samples of unphosphorylated and phosphorylated eIF2 are provided as references. The prominent band at ~70 kDa present in all lanes is bovine serum albumin (BSA), utilised as a stabiliser in all reactions (it obscures the GST-PERK signal, where applicable). Migration of eIF2B subunits is indicated on the left.

B) Upper right panel: Plot of time-dependent change in fluorescence polarisation of FAM-ISRIB bound to wildtype eIF2B in presence or absence of phosphorylated eIF2. Where indicated, at t = 0 a specific eIF2(αP)-directed holophosphatase consisting of G-actin/PP1A/PPP1R15A (GAP) or the non-specific lambda phosphatase (λP) was introduced to convert phosphorylated eIF2 to eIF2. Shown is a representative experiment (one of two).

Lower right panel: Coomassie-stained PhosTag SDS-PAGE gel of the samples analysed in the experiment above. The eIF2 subunit, including phosphorylated and unphosphorylated eIF2α, are indicated on the right, eIF2B subunits and species arising from the phosphatase-treated samples are indicated on the left (the catalytic subunit PP1A is not visible on this gel; the asterisk marks an unidentified contaminant of the λP samples).

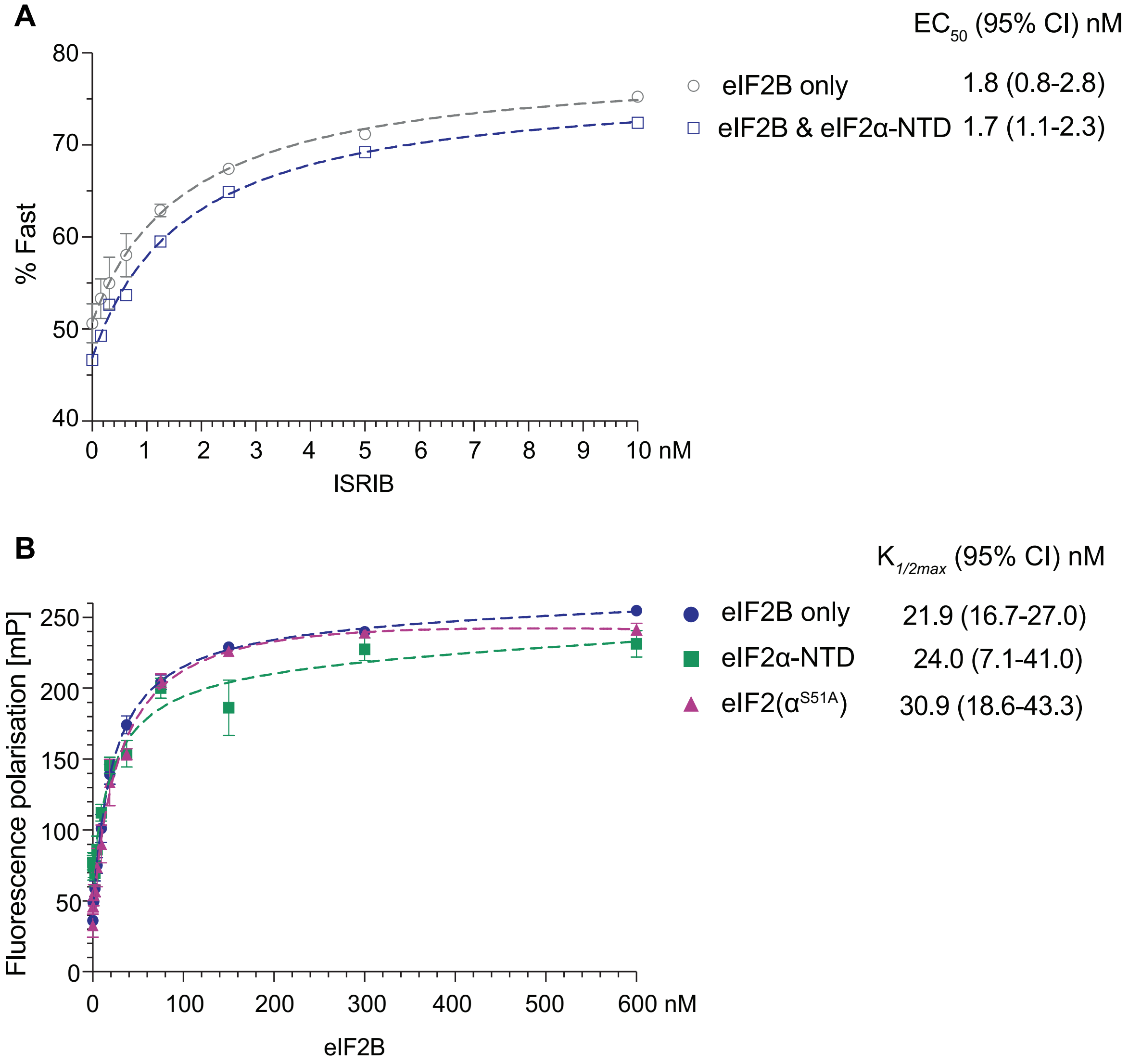

Supplementary Figure 4. Lack of cooperativity between unphosphorylated eIF2 and ISRIB in binding to eIF2B, related to Figure 5.

A) Unphosphorylated eIF2α-NTD does not affect the dissociation of eIF2B from P-eIF2α-NTD in the presence of ISRIB. As in Figure 5C (right plot): plot of the %Fast of the dissociation reactions as a function of ISRIB concentration, obtained through BLI experiment monitoring dissociation of eIF2B from immobilised P-eIF2α-NTD in the presence of indicated concentrations of ISRIB. The grey curve (eIF2B only) is redrawn from Figure 5C (right plot), the blue curve indicates the dissociation performed in the presence of unphosphorylated eIF2α-NTD. The data was fitted to an [Agonist] vs. response (Hill slope = 1) non-linear regression model (dotted line). EC_{50} with 95% CI is indicated.

B) Presence of unphosphorylated eIF2 does not affect binding of FAM-ISRIB to eIF2B. Plot of fluorescence polarisation signals (mean ± SD, n=3) arising from samples of FAM-conjugated ISRIB (2.5 nM) incubated with varying concentrations of wildtype eIF2B. Where indicated 15 μM eIF2α-NTD or 1 μM eIF2(α^{S51A}) was added. $K_{1/2max}$ with 95% CI is shown. The difference in $K_{1/2max}$ values in these experiments, compared with those shown in Figure 3A, likely reflect differences in eIF2B preparations.

Table S1. List of cell lines, related to Figure 1C & D, and Figure 6.

Gene	Exon	Cells	Clone name	Description	Mutagenized region (numbers indicate amino acid position at which mutagenesis occurred)
NA	NA	CHO-S21	NA	dual reporter [CHOP::GFP; Xbp1::Turquoise] parental cell line from Sekine et al. 2016	NA
Eif2S1	2	CHO S51A	NA	dual reporter ISR-insensitive (gcn-) eIF2α^{S51A} from Sekine et al. 2016	51_**A**RRRIRSI
Eif2b4	10	CHO-S21	12H6	eIF2Bδ(L316N), ISR-insensitive	316_**N**AAQAISRF
Eif2b4	10	CHO-S21	22H2	eIF2Bδ(E312K; L316V), ISR-insensitive	312_**K**KIV_316_**V**AAQA
Eif2b3	11	CHO-C30	S7	CHOP::GFP-reporter eIF2Bγ-3xFlag-tagged cells from Zyryanova et al. 2018	451_EFCRYPAQWRPLERADYKDHDGDYKDHDIDYKDDDDK*
EIF2B2	1	HeLa	2C2	3 X Flag-tagged eIF2Bβ cells from Sekine et al. 2015	2_PGSDYKDHDGDYKDHDIDYKDDDDK
FreeStyle 293-F cells	NA	HEK293	NA	Mammalian expression cell line	NA

Table S2. List of plasmids, related to Figures as indicated.

ID	Plasmid name	Description	Primers used to generate plasmid	Figures
UK2320	CHO_EIF2B4_EXON10_g3_pSpCas9(BB)-2A-Puro	CRISPR/ Cas9 with puromycin selection targeting hamster *Eif2b4* (eIF2B delta) gene	Oligo 2209 & 2210	1C
UK2733	heIF2a_2-187_WT_AviTag_H6_pET-30a(+)	wildtype NTD human eIF2alpha_1-187 with AviTag and 6x histidines in bacterial expression vector	NA	3B
UK1610	pSpCas9(BB)-2A-mCherry_V2	CRISPR/ Cas9 empty vector with mCherry selection	NA	5A
UK2536	cgeIF2B2_g2_pSpCas9(BB)-2A-mCherry	CRISPR/ Cas9 with mCherry selection targeting hamster *Eif2b2* (eIF2B beta) gene (guide 1)	Oligo 2520 & 2521	5A
UK2537	cgeIF2B2_g3_pSpCas9(BB)-2A-mCherry	CRISPR/ Cas9 with mCherry selection targeting hamster *Eif2b2* (eIF2B beta) gene (guide 2)	Oligo 2522 & 2523	5A
UK2538	cgeIF2B4_g1_pSpCas9(BB)-2A-mCherry	CRISPR/ Cas9 with mCherry selection targeting hamster *Eif2b4* (eIF2B delta) gene (guide 1)	Oligo 2524 & 2525	5A
UK2539	cgeIF2B4_g3_pSpCas9(BB)-2A-mCherry	CRISPR/ Cas9 with mCherry selection targeting hamster *Eif2b4* (eIF2B delta) gene (guide 2)	Oligo 2526 & 2527	5A
UK2547	cgeIF2B5_g1_pSpCas9(BB)-2A-mCherry	CRISPR/ Cas9 with mCherry selection targeting hamster *Eif2b5* (eIF2B epsilon) gene	Oligo 2543 & 2544	5A
UK1367	pSpCas9(BB)-2A-Puro	CRISPR/ Cas9 empty vector with puromycin selection	NA	5C
UK1505	CHO_Eif2s1_guideA_pSpCas9(BB)-2A-Puro	CRISPR/ Cas9 with puromycin selection targeting hamster *Eif2s1* (eIF2 alpha) gene (guide A)	Oligo 1015 & 1018	5C
UK1506	CHO_Eif2s1_guideB_pSpCas9(BB)-2A-Puro	CRISPR/ Cas9 with puromycin selection targeting hamster *Eif2s1* (eIF2 alpha) gene (guide B)	Oligo 1016 & 1019	5C
UK2731	heIF2a_2-187_pSUMO3	encodes H6-SUMO3-SER_hueIF2a_2-187	NA	S4A
NA	*pET28-3C-2B1*	For bacterial expression of eIF2B alpha from Kashiwagi et al. 2019	NA	1A&B, 2-5, S1, S2
NA	*pETDuet-2B4-2B2*	For bacterial expression of eIF2B delta and beta from Kashiwagi et al. 2019	NA	1A&B, 2-5, S1-2
NA	*pCOLADuet-2B5-2B3*	For bacterial expression of eIF2B epsilon and gamma from Kashiwagi et al. 2019	NA	1A&B, 2-5, S1-2
NA	*pETDuet-2B4-2B2_dE310K*	For bacterial expression of eIF2B delta-E310K and beta	NA	1B, 3A&C, 4B
NA	*pETDuet-2B4-2B2_dL314Q*	For bacterial expression of eIF2B delta-L314Q and beta	NA	1B, 3A&C, 4B
NA	*pEBMulti-Neo-human-eIF2alpha*	For mammalian expression of eIF2 alpha from Kashiwagi et al. 2019	NA	2, S1-2
NA	*pEBMulti-Neo-human-eIF2alpha-PA*	For mammalian expression of eIF2 alpha	NA	3B&C, 4B, S3
NA	*pEBMulti-Neo-human-eIF2alpha-S52A-PA*	For mammalian expression of eIF2 alpha-S51A	NA	1A&B, 4B, S4B
NA	*pEBMulti-Neo-human-eIF2beta*	For mammalian expression of eIF2 beta from Kashiwagi et al. 2019	NA	1A&B, 2, 3B&C, 4B, S1-3, S4B
NA	*pEBMulti-Neo-human-eIF2gamma-FlagHis8*	For mammalian expression of eIF2 gamma from Kashiwagi et al. 2019	NA	1A&B, 2, 3B&C, 4B, S1-3, S4B

Table S3. List of primers, related to Figures as indicated.

ID	Oligo name	Sequence	Description	Figures
Oligo 2209	CHO_EIF2B4_EXON10_g3_1s	CACCGAAGATTGTGCTTGCAGCTC	sense primer to create UK2320 with sgRNA targeting hamster *Eif2b4* gene	1C
Oligo 2210	CHO_EIF2B4_EXON10_g3_2AS	AAACGAGCTGCAAGCACAATCTTC	anti-sense to create UK2320 with sgRNA targeting hamster *Eif2b4* gene	1C
Oligo 2213	CHO_eIF2B4_Exon10_ssODN_L310X	GGTTTTTCAGTCAGGTATTCACCATACCATCCATATACCAGGATCACGTCCCCGTCACTGATCTTCTTAGAGGCAAACCGTGAAATTGCTTGAGCTGC**NNN**CACAATCTTCTCTTGTACATACCGATCAATGGCTTCTCTAAGTTCTGACTTTGCCTAAATGTTGAGAGAACAGTGATATAATTCACCC	single strand ODN repair template introducing eIF2Bδ(L316N), ISR-insensitive phenotype	1C
Oligo 2214	CHO_eIF2B4_Exon10_ssODN_E306K_L310X	GGTTTTTCAGTCAGGTATTCACCATACCATCCATATACCAGGATCACGTCCCCGTCACTGATCTTCTTAGAGGCAAACCGTGAAATTGCTTGAGCTGC**NNN**CACAATCTTCTTTTGTACATACCGATCAATGGCTTCTCTAAGTTCTGACTTTGCCTAAATGTTGAGAGAACAGTGATATAATTCACCC	single strand ODN repair template introducing eIF2Bδ(E312K; L316V), ISR-insensitive phenotype	1C
Oligo 2520	cgeIF2B2_g2_S	CACCGCACACTCGGCAACAATGACA	sense primer to create UK2536 with sgRNA targeting hamster *Eif2b2* gene (guide 1)	5A
Oligo 2521	cgeIF2B2_g2_AS	AAACTGTCATTGTTGCCGAGTGTGC	anti-sense to create UK2536 with sgRNA targeting hamster *Eif2b2* gene (guide 1)	5A
Oligo 2522	cgeIF2B2_g3_S	CACCGATGGGTGCACACACGATGAG	sense primer to create UK2537 with sgRNA targeting hamster *Eif2b2* gene (guide 2)	5A
Oligo 2523	cgeIF2B2_g3_AS	AAACCTCATCGTGTGTGCACCCATC	anti-sense to create UK2537 with sgRNA targeting hamster *Eif2b2* gene (guide 2)	5A
Oligo 2524	cgeIF2B4_g1_S	CACCGATTATGCGCTCGAGCTACGA	sense primer to create UK2538 with sgRNA targeting hamster *Eif2b4* gene (guide 1)	5A
Oligo 2525	cgeIF2B4_g1_AS	AAACTCGTAGCTCGAGCGCATAATC	anti-sense to create UK2538 with sgRNA targeting hamster *Eif2b4* gene (guide 1)	5A

Oligo 2526	cgeIF2B4_g3_S	CACCGGAACCGCCTGCCCTCGACCC	sense primer to create UK2538 with sgRNA targeting hamster *Eif2b4* gene (guide 2)	5A
Oligo 2527	cgeIF2B4_g3_AS	AAACGGGTCGAGGGCAGGCGGTTCC	anti-sense to create UK2538 with sgRNA targeting hamster *Eif2b4* gene (guide 2)	5A
Oligo 2543	cgeIF2B5_g1_S	CACCGGAACAAAATCATCTCGAGTT	sense primer to create UK2547 with sgRNA targeting hamster *Eif2b5* gene	5A
Oligo 2544	cgeIF2B5_g1_AS	AAACAACTCGAGATGATTTTGTTCC	anti-sense to create UK2547 with sgRNA targeting hamster *Eif2b5* gene	5A
Oligo 1015	CHO_eif2s1_CrispyA_1s	CACCGTATTCCAACAAGCTAACAT	sense primer to create UK1505 with sgRNA targeting hamster *Eif2s1* gene (guide A)	5C
Oligo 1018	CHO_eif2s1_CrispyA_2AS	AAACATGTTAGCTTGTTGGAATAC	anti-sense primer to create UK1505 with sgRNA targeting hamster *Eif2s1* gene (guide A)	5C
Oligo 1016	CHO_eif2s1_CrispyB_1s	CACCGGGAGCCTATGTTAGCTTGT	sense primer to create UK1506 with sgRNA targeting hamster *Eif2s1* gene (guide B)	5C
Oligo 1019	CHO_eif2s1_CrispyB_2AS	AAACACAAGCTAACATAGGCTCCC	anti-sense primer to create UK1506 with sgRNA targeting hamster *Eif2s1* gene (guide B)	5C
