# Supplementary material for: ISRIB Blunts the Integrated Stress Response by Allosterically Antagonising the Inhibitory Effect of Phosphorylated eIF2 on eIF2B
Source: Mol Cell. 2021 Jan 7;81(1):88–103.e6. doi: 10.1016/j.molcel.2020.10.031 (PMC7837216; doi:10.1016/j.molcel.2020.10.031)
Supplement: Document S2. Article plus Supplemental Information [file mmc2.pdf]

Molecular Cell

Article

ISRIB Blunts the Integrated Stress Response by Allosterically Antagonising the Inhibitory Effect of Phosphorylated eIF2 on eIF2B

Graphical Abstract

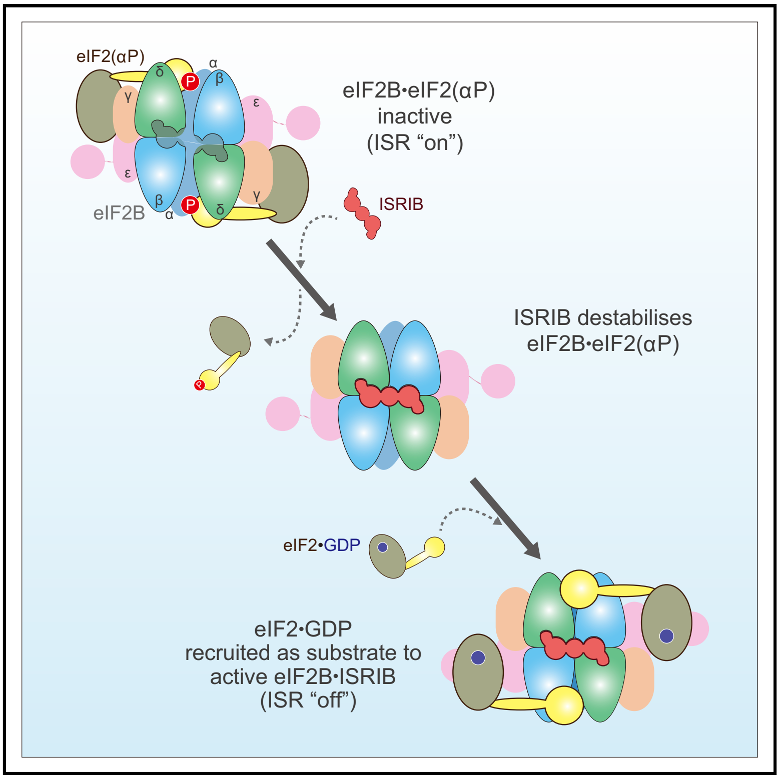

Authors

Alisa F. Zyryanova,
Kazuhiro Kashiwagi, Claudia Rato, ...,
Mikako Shirouzu, Takuhiro Ito,
David Ron

Correspondence

takuhiro.ito@riken.jp (T.I.),
dr360@medschl.cam.ac.uk (D.R.)

In Brief

ISRIB is a drug-like compound that restores memory and cognitive function in mouse models of certain human diseases. Its beneficial properties derive from interference with a stress-response pathway that hinges on regulating rates of protein synthesis. Here, Zyryanova, Kashiwagi et al. dissect the molecular basis of ISRIB action in cells.

Highlights

- Mutually antagonistic binding of phosphorylated eIF2 and ISRIB to eIF2B
- Opposing structural rearrangement of eIF2B by binding ISRIB or phosphorylated eIF2
- Phosphorylated eIF2 exposes ISRIB's ability to enhance eIF2B activity *in vitro*
- Weak ISR-inhibitory activity of ISRIB in cells lacking phosphorylated eIF2

Zyryanova et al., 2021, Molecular Cell *81*, 88–103
January 7, 2021 © 2020 The Author(s). Published by Elsevier Inc.
https://doi.org/10.1016/j.molcel.2020.10.031

CellPress
OPEN ACCESS

Molecular Cell

Article

ISRIB Blunts the Integrated Stress Response by Allosterically Antagonising the Inhibitory Effect of Phosphorylated eIF2 on eIF2B

Alisa F. Zyryanova,[1,3] Kazuhiro Kashiwagi,[2,3] Claudia Rato,[1] Heather P. Harding,[1] Ana Crespillo-Casado,[1] Luke A. Perera,[1] Ayako Sakamoto,[2] Madoka Nishimoto,[2] Mayumi Yonemochi,[2] Mikako Shirouzu,[2] Takuhiro Ito,[2,4,*] and David Ron[1,4,5,*]
[1]Cambridge Institute for Medical Research (CIMR), University of Cambridge, Cambridge CB2 0XY, UK
[2]RIKEN Center for Biosystems Dynamics Research, Suehiro-cho, Tsurumi-ku, Yokohama 230-0045, Japan
[3]These authors contributed equally
[4]Senior author
[5]Lead Contact
*Correspondence: takuhiro.ito@riken.jp (T.I.), dr360@medschl.cam.ac.uk (D.R.)
https://doi.org/10.1016/j.molcel.2020.10.031

SUMMARY

The small molecule ISRIB antagonizes the activation of the integrated stress response (ISR) by phosphorylated translation initiation factor 2, eIF2(αP). ISRIB and eIF2(αP) bind distinct sites in their common target, eIF2B, a guanine nucleotide exchange factor for eIF2. We have found that ISRIB-mediated acceleration of eIF2B's nucleotide exchange activity *in vitro* is observed preferentially in the presence of eIF2(αP) and is attenuated by mutations that desensitize eIF2B to the inhibitory effect of eIF2(αP). ISRIB's efficacy as an ISR inhibitor in cells also depends on presence of eIF2(αP). Cryoelectron microscopy (cryo-EM) showed that engagement of both eIF2B regulatory sites by two eIF2(αP) molecules remodels both the ISRIB-binding pocket and the pockets that would engage eIF2α during active nucleotide exchange, thereby discouraging both binding events. *In vitro*, eIF2(αP) and ISRIB reciprocally opposed each other's binding to eIF2B. These findings point to antagonistic allostery in ISRIB action on eIF2B, culminating in inhibition of the ISR.

INTRODUCTION

Under diverse stressful conditions, the α-subunit of eukaryotic translation initiation factor 2 (eIF2) is phosphorylated on serine 51 in its N-terminal domain (NTD). This converts eIF2, the substrate of eIF2B, to an inhibitor of eIF2B; a guanine nucleotide exchange factor (GEF) that reactivates the eIF2 heterotrimer by accelerating the release of GDP from the γ-subunit and its exchange with GTP (Ranu and London, 1979; de Haro et al., 1996), thus promoting binding of initiator methionyl-tRNA (Met-$tRNA_i$) to eIF2·GTP (Dev et al., 2010). By depleting ternary complexes of eIF2, GTP, and Met-$tRNA_i$ in the cell, eIF2α phosphorylation attenuates the translation of most mRNAs, with important effects on protein synthesis. However, translation of few mRNAs is increased in an eIF2 phosphorylation-dependent manner. As the latter encode potent transcription factors, the production of phosphorylated eIF2 [eIF2(αP)] is coupled with a conserved gene expression program referred to as the integrated stress response (ISR) (Harding et al., 2003).

The ISR is a homeostatic pathway that contributes to organismal fitness (Pakos-Zebrucka et al., 2016). However, in some circumstances, its heightened activity is associated with unfavorable outcomes, motivating a search for ISR inhibitors. When applied to cells or administered to animals, the drug-like small molecule, ISRIB, disrupts the ISR (Sidrauski et al., 2013) and has been reported to exert beneficial effects in models of neurodegeneration (Halliday et al., 2015; Zhu et al., 2019), head injury (Chou et al., 2017), and dysmyelination (Wong et al., 2018; Abbink et al., 2019).

ISRIB does not affect the levels of eIF2(αP), indicating a site of action downstream of this common effector. ISRIB-resistant mutations were mapped genetically to the β- and δ-subunits of eIF2B and disrupt the high-affinity binding of ISRIB (K_d ~10 nM) to a pocket on the surface of eIF2B (Sekine et al., 2015; Tsai et al., 2018; Zyryanova et al., 2018), demonstrating that eIF2B is ISRIB's target.

eIF2B is an ~500 kDa decamer, assembled from two sets of five subunits (Kashiwagi et al., 2016). It has two catalytic sites, each comprised of the bipartite ε-subunit whose two domains embrace the nucleotide-binding eIF2γ enforcing a conformation that favors GDP dissociation and exchange with GTP. Engagement of unphosphorylated eIF2 in this catalytically productive conformation depends on binding of the NTD of the eIF2 α-subunit (eIF2α-NTD) in a pocket between the β- and δ-subunits of eIF2B, ~100 Å from the catalytic site (Kashiwagi et al., 2019; Kenner et al., 2019). When eIF2 is phosphorylated, the

 © 2020 The Author(s). Published by Elsevier Inc.
This is an open access article under the CC BY license (http://creativecommons.org/licenses/by/4.0/).

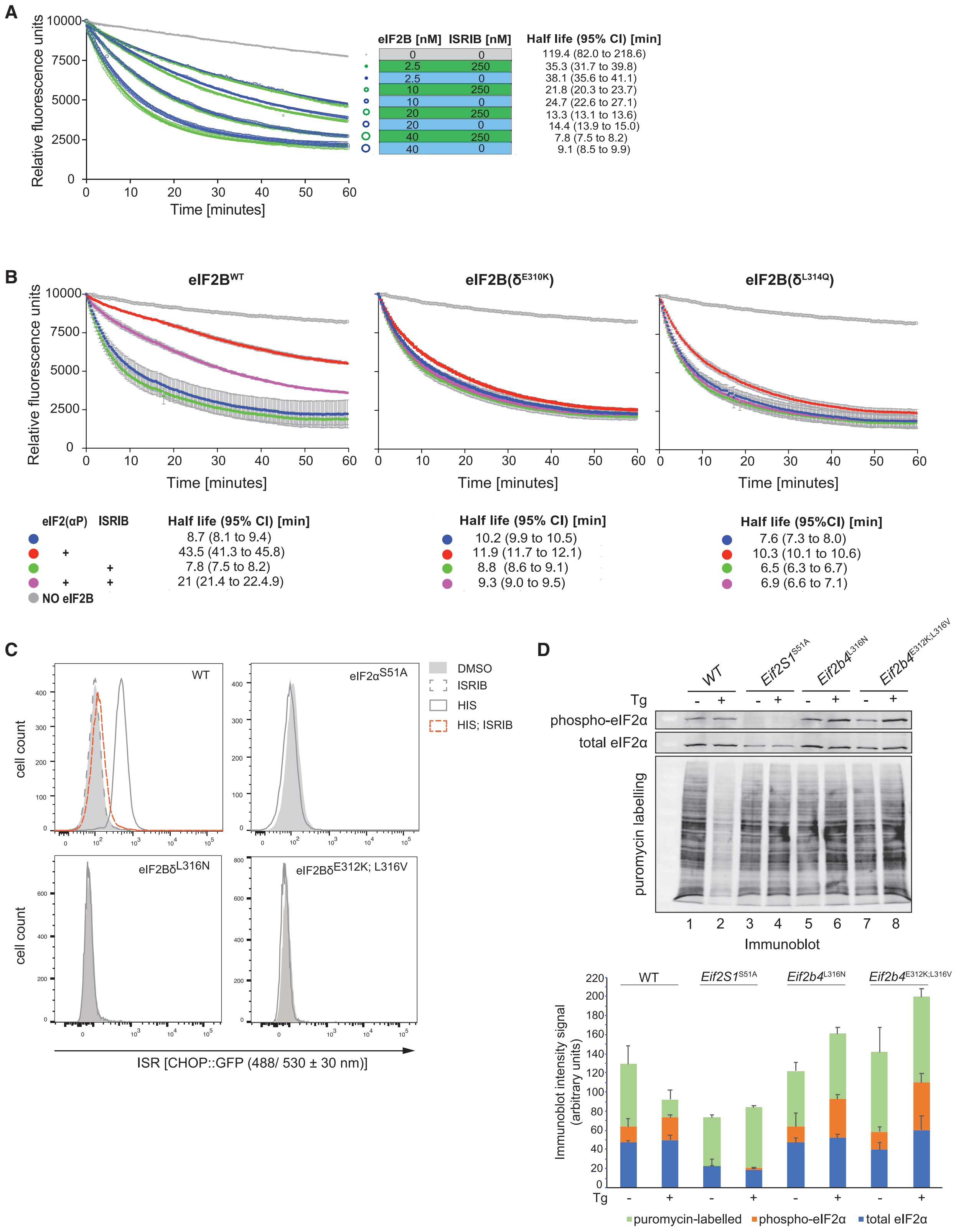

(legend on next page)

phosphorylated eIF2α-NTD (P-eIF2α-NTD) engages eIF2B at an alternative site, between the α- and δ-subunits of eIF2B (Adomavicius et al., 2019; Gordiyenko et al., 2019; Kashiwagi et al., 2019; Kenner et al., 2019): a catalytically nonproductive binding mode that inhibits eIF2B's nucleotide exchange activity (Kashiwagi et al., 2019). ISRIB binds a single and distinct site on eIF2B, at its center of symmetry, the interface between the β- and δ-subunits of eIF2B (Tsai et al., 2018; Zyryanova et al., 2018) (see Figure 7A, below).

It stands to reason that ISRIB inhibits the ISR by promoting the nucleotide exchange activity of eIF2B. Indeed, when added to crude preparations of eIF2B, ISRIB accelerates exchange of GDP nucleotide on its substrate eIF2 (Sekine et al., 2015; Sidrauski et al., 2015). A simple mechanism has been proposed to account for such stimulation: decameric eIF2B consists of one α_2 dimer and two βδγε tetramers; by binding across the interface between the two tetramers, ISRIB favors decamer assembly and stability. According to this model, which is well supported by features of eIF2B's assembly *in vitro*, ISRIB inhibits the ISR by increasing the effective concentration of active, decameric eIF2B (Tsai et al., 2018).

Accelerated assembly of eIF2B as ISRIB's mode of action would be favored by the presence of a large pool of unassembled eIF2B subunits in the cell. Yet, fractionation of mammalian cell lysates by density gradient centrifugation has not suggested the existence of large pools of precursor complexes of eIF2B subunits (Sidrauski et al., 2015; Zyryanova et al., 2018). Being a slow fractionation method, density gradient centrifugation might fail to detect a pool of precursors migrating at their predicted position in the gradient, if the precursors were in a rapid equilibrium with the assembled decamers. However, the finding that ISRIB has little to no effect on the nucleotide exchange activity of pure eIF2B decamers (Tsai et al., 2018) speaks against ISRIB increasing active enzyme concentration by stabilizing the decamer in such a rapid equilibrium.

These considerations, and hints of structural differences in the conformation of the eIF2α-binding pocket on eIF2B between the productive and nonproductive complexes (Gordiyenko et al., 2019; Kashiwagi et al., 2019; Kenner et al., 2019), prompted us to examine the evidence for alternative modes of ISRIB action. Here, we report on biochemical, structural, and cell-based findings that ISRIB allosterically antagonizes the inhibitory effect of eIF2(αP) on eIF2B's guanine nucleotide exchange activity to inhibit the ISR.

RESULTS

ISRIB Accelerates eIF2B Guanine Nucleotide Exchange Activity in Presence of eIF2(αP)

To assess the effects of ISRIB on eIF2B guanine nucleotide exchange activity in isolation of phosphorylated eIF2, we loaded BODIPY-GDP onto eIF2(α^{S51A}) isolated from 293-F cells expressing eIF2 with a non-phosphorylatable S51A mutation in the α-subunit. We then utilized eIF2(α^{S51A}) as a substrate in a fluorescence-based nucleotide exchange assay with recombinant human eIF2B. As reported previously (Tsai et al., 2018), ISRIB only minimally accelerated the exchange of nucleotide mediated by eIF2B in an assay devoid of eIF2(αP) (Figure 1A). Introduction of eIF2(αP) into the assay attenuated the nucleotide exchange activity directed toward the non-phosphorylatable eIF2(α^{S51A})·BODIPY-GDP. This effect was significantly, although only partially, reversed by ISRIB (Figure 1B), as observed previously (Wong et al., 2018).

The inhibitory effect of eIF2(αP) observed *in vitro* was attenuated by mutations in human eIF2Bδ residues, E310K and L314Q (Figure 1B), as reported previously (Kimball et al., 1998). When introduced into the genome of cultured CHO cells (by CRISPR/Cas9-mediated homologous recombination), mutations in the corresponding hamster residues (eIF2Bδ E312 and L316) imparted an ISR-insensitive phenotype, as reflected in the blunted stress-induced activation of the ISR-responsive CHOP::GFP reporter gene (Figure 1C) and in the blunted repression of protein synthesis, normally observed in stressed cells (Figure 1D). These findings are consistent with the phenotype of corresponding mutations in yeast, GCD2^{E377K} and GCD2^{L381Q} (Pavitt et al., 1997). *In vitro*, equivalent substitutions in human eIF2Bδ, E310K and L314Q also blunted the response to ISRIB that was observed with wild-type eIF2B in presence of eIF2(αP) (Figure 1B). Together, these observations indicate that, *in vitro*, ISRIB reverses an inhibitory effect of eIF2(αP) on the guanine nucleotide exchange activity of eIF2B that is relevant to the activation of the ISR *in vivo*.

eIF2(αP) Induces an eIF2B Conformation Inimical to ISRIB Binding

The recently published structures of eIF2·eIF2B complexes (Adomavicius et al., 2019; Gordiyenko et al., 2019; Kashiwagi et al., 2019; Kenner et al., 2019) suggest that binding of eIF2(αP) alters the conformation of eIF2B. However, their resolution limits

Figure 1. ISRIB Accelerates eIF2B Guanine Nucleotide Exchange Activity Selectively in Presence of eIF2(αP)

(A) Plots of eIF2B guanine nucleotide exchange activity as reflected in time-dependent decrease in fluorescence of BODIPY-FL-GDP bound to non-phosphorylatable eIF2(α^{S51A}) (125 nM). Blue traces lack and green traces include ISRIB (250 nM), the gray trace lacks eIF2B. The size of the symbol reflects the concentration of eIF2B in the assay. All the data points of a representative experiment performed in duplicate are shown. The half-life of GDP binding with 95% confidence interval (CI) for each plot is indicated (observation reproduced three times).

(B) As in (A) but utilizing a fixed concentration of wild-type or the indicated ISR-insensitive eIF2B mutants (40 nM), BODIPY-FL-GDP bound to non-phosphorylatable eIF2(α^{S51A}) (125 nM) and where indicated, eIF2(αP) (1 μM) and ISRIB (250 nM). Plotted are the mean fluorescence values ± SD of an experiment performed in technical triplicate.

(C) *In vivo* characterization of the ISR in wild-type and mutant CHO cells of the indicated genotype. Shown are histograms of the activity of CHOP::GFP (an ISR reporter gene) in untreated cells and cells in which the eIF2α kinase GCN2 had been activated by L-histidinol in the absence or presence of ISRIB. Shown is a representative experiment reproduced three times.

(D) Estimates of protein synthesis rates in CHO cells of the indicated genotype before and after activation of the eIF2α kinase PERK by thapsigargin (Tg). The lower panel is an anti-puromycin immunoblot of whole cell lysates, in which the intensity of the puromycinylated protein signal reports on rates of protein synthesis. The upper panels are immunoblots of P-eIF2α and of total eIF2α. Below is a stacked column graph of the quantified blot signals (mean ± SD, n = 3): puromycin-labeled in light green (top), P-eIF2α in orange (middle), and total eIF2α in blue (bottom).

Table 1. Cryo-EM Data Collection and Image Processing

	α^P1 complex (EMDB-30570) (PDB 7D45)	α^P2 complex (EMDB-30569) (PDB 7D44)	$\alpha^P\gamma$ complex (EMDB-30568) (PDB 7D43)	eIF2B apo (EMDB-30571) (PDB 7D46)
Data collection and processing				
Microscope	Tecnai Arctica			Tecnai Arctica
Camera	K2 Summit			K2 Summit
Magnification	23,500			23,500
Voltage (kV)	200			200
Electron exposure ($e^-/Å^2$)	50			50
Exposure per frame	1.25			1.25
Number of frames collected	40			40
Defocus range (μm)	−1.5 to −3.1			−1.5 to −3.1
Micrographs	7,729			4,987
Pixel size (Å)	1.47			1.47
3D Processing package	RELION-3.0			RELION-2.1,3.0
Symmetry imposed	C1			C1
Initial particle images	1,889,101			1,482,123
Final particle images	208,728	80,921	66,721	330,601
Initial reference map	5B04 (40 Å)	5B04 (40 Å)	5B04 (40 Å)	5B04 (40 Å)
Map resolution				
Masked (FSC = 0.143)	3.77	4.01	4.28	3.97
Map sharpening B-factor	−119.0	−103.6	−108.8	−165.6
Refinement				
Initial model used	6O9Z, 6K72	6O9Z, 6K72	6O9Z, 6K72	6O9Z, 6K72
Model composition				
Non-hydrogen atoms	27,529	28,390	31,005	26,636
Protein residues	3,659	3,825	4,347	3,481
B factors				
Protein	26.74	37.47	71.83	23.55
RMSD				
Bond lengths (Å)	0.009	0.006	0.006	0.008
Bond angles (°)	0.916	0.850	0.975	0.928
Validation				
MolProbity score	2.48	2.48	2.64	2.47
Clashscore	19.53	20.16	27.64	18.44
Poor rotamers (%)	0.35	0.28	0.31	0.42
CaBLAM outliers (%)	5.78	5.19	6.79	6.08
Ramachandran plot				
Favored (%)	83.13	83.68	81.65	82.43
Allowed (%)	16.76	16.26	18.30	17.52
Disallowed (%)	0.11	0.05	0.05	0.06
Map CC (CC_{mask})	0.80	0.78	0.79	0.82

See also Figure S1.

confidence in this conclusion, especially in regard to the ISRIB-binding site. Therefore, we collected further cryo-electron microscopy (cryo-EM) images of human eIF2B in complex with the eIF2(αP) trimer and solved three new variant structures: complexes between eIF2B and one eIF2(αP) trimer in which only the α-subunit is resolved (the α^P1 complex), complexes with two eIF2(αP) trimers in which two molecules of the eIF2 α-subunit are resolved at both ends of eIF2B (α^P2 complex), and complexes with two eIF2(αP) trimers in which both the α- and γ-subunits are resolved on one end of eIF2B and only the eIF2 α-subunit is resolved on the other end of eIF2B (the $\alpha^P\gamma$ complex) (Table 1; Figure S1). The overall binding modes of eIF2(αP) in

these structures are similar to those previously observed (Adomavicius et al., 2019; Gordiyenko et al., 2019; Kashiwagi et al., 2019; Kenner et al., 2019), and the subunits' conformation in the $\alpha^P\gamma$ complex are almost identical to those in previous structures (Kashiwagi et al., 2019), but with improved resolution. In addition, we re-analyzed the previous dataset for the eIF2B·eIF2(αP) complex (Kashiwagi et al., 2019) and extracted the apo eIF2B particles (Table 1; Figure S1).

When the apo eIF2B and $\alpha^P\gamma$ complex structures are compared, the binding of eIF2(αP) is associated with an en bloc rearrangement of the βδγε tetrameric unit of eIF2B (Figure 2A). This widens the gap between eIF2Bβ and eIF2Bδ that would otherwise accommodate the eIF2α-NTD in the catalytically productive conformation (Figures 2B and S2A) (Kashiwagi et al., 2019; Kenner et al., 2019). As a consequence, the C^α atoms of eIF2Bδ helix α3 (helix δ-α3, residues 247–267), which intensively interact with the eIF2α-NTD at the eIF2Bδ-side of the gap, are displaced 3.2 Å (on average) and its helical axis is rotated 7.9° away from eIF2Bβ (the root-mean-square deviation [RMSD] for the alignment of eIF2Bβ is 0.7 Å) (Figure 2B, right panel). A similar widening of the gap is also observed in the α^P2 complex (average displacement of helix δ-α3 is 2.8 Å and its rotation is 8.1°) (Figures S2A and S2B).

The arrangement of the gap in apo eIF2B is more similar to the structure that accommodates the unphosphorylated eIF2α-NTD (PDB: 6O81) (Kenner et al., 2019) with only a minor further narrowing of the gap observed following the accommodation (the average displacement of helix δ-α3 is 1.1 Å and its rotation is 2.3° toward eIF2Bβ) (Figures 2B and S2A). This suggests that widening of the gap observed upon binding of eIF2(αP) antagonizes catalytically productive binding of eIF2. Such widening of the gap between eIF2Bβ and eIF2Bδ appears to be a conserved feature, because the gaps in the structures of yeast eIF2B bound by two eIF2(αP) trimers are wider than the human $\alpha^P\gamma$ complex (Gordiyenko et al., 2019). Widening of the gap between eIF2Bβ and eIF2Bδ was previously observed in the structure of eIF2B complexed with the isolated phosphorylated eIF2 α-subunit (P-eIF2α) (PDB: 6O9Z) (Kenner et al., 2019), but is more conspicuous in the presently determined $\alpha^P\gamma$ complex structure (the average displacement of helix δ-α3 is 2.4 Å in 6O9Z versus 3.2 Å in the $\alpha^P\gamma$ complex) (Figure S2B). Therefore, the γ-subunit of eIF2(αP) seems to make some additional contribution to this structural rearrangement of the subunits of eIF2B. Contacts between the γ-subunit of eIF2(αP) and eIF2Bγ observed in the $\alpha^P\gamma$ complex may contribute to this difference, but their significance needs further exploration (Figures 2A and S2A).

The rearrangement of eIF2B induced by eIF2(αP) also affects the pocket for ISRIB. This pocket is formed by two heterodimeric units of eIF2Bβ and eIF2Bδ (Tsai et al., 2018; Zyryanova et al., 2018). Comparing the $\alpha^P\gamma$ complex with the eIF2B·ISRIB complex (PDB: 6CAJ) (Tsai et al., 2018) reveals that the relative arrangement of these two heterodimeric units is altered. C^α atoms around ISRIB (within 10 Å) of one βδ unit (eIF2Bβ and eIF2Bδ in Figure 2C) are displaced on average 2.2 Å away from the other β′δ′ unit (eIF2Bβ′ and eIF2Bδ′ in Figure 2C) in $\alpha^P\gamma$ complex. This displacement includes key residues involved in ISRIB action and binding (Figures 2C and S2C) (Sekine et al., 2015; Tsai et al., 2018; Zyryanova et al., 2018). A similar displacement is also observed in the α^P2 complex (Figure S2D). The binding of ISRIB thus fixes the relative arrangement of these two heterodimeric units, favoring the conformation observed in the productive enzyme-substrate complex and disfavoring the nonproductive rearrangement that accommodates eIF2(αP).

Compared to the $\alpha^P\gamma$ and α^P2 complexes that contain two eIF2(αP) trimers, the rearrangement observed in the α^P1 complex, which contains only one eIF2(αP) trimer, is subtler. Although the eIF2Bα_2 homodimeric unit is displaced following the accommodation of eIF2(αP) (Figure S2E), there are negligible shifts in the other parts of eIF2B, including the regulatory cleft between eIF2Bβ and eIF2Bδ (the average displacement of helix δ-α3 is 0.6 Å) and the pocket for ISRIB (the relative displacement between the βδ heterodimeric units is 0.4 Å) (Figures 2B and S2D). Therefore, the aforementioned rearrangement induced by eIF2(αP) was accentuated by accommodation of the second eIF2(αP) trimer. The coupling between eIF2(αP) binding at its regulatory sites and the progressive deformation of the ISRIB-binding pocket brought about by sequential binding of two eIF2(αP) trimers sets the stage for a competition, whereby ISRIB-mediated stabilization of its pocket is propagated in a reciprocal manner to the eIF2(αP)-binding sites. ISRIB is expected to be especially antagonistic toward engagement of a second eIF2(αP) trimer, hence discouraging eIF2B from assuming its most inhibited conformation.

By contrast, the structural interplay between the binding of ISRIB and unphosphorylated eIF2 is inconspicuous. Co-binding of ISRIB and unphosphorylated eIF2 to eIF2B has been observed (Kenner et al., 2019). In addition, the aforementioned drawing together of eIF2Bβ and eIF2Bδ around the unphosphorylated eIF2 is observed in the presence or absence of ISRIB (PDB: 6K71) (Kashiwagi et al., 2019), whereas no movement is induced by the binding of ISRIB alone (PDB: 6CAJ) (Tsai et al., 2018) (Figure S2B). These structural considerations suggest that the binding of ISRIB is unlikely to contribute to eIF2B's affinity toward the unphosphorylated eIF2. Furthermore, neither the binding of ISRIB nor unphosphorylated eIF2 induce observable rearrangement between two eIF2Bβ-eIF2Bδ heterodimeric units at the ISRIB-binding pocket (the average movement between the βδ heterodimeric units upon individual binding of ISRIB and unphosphorylated eIF2 are 0.3 Å and 0.1 Å, respectively) (Figure S2D, lower panel). On structural grounds alone, the binding of ISRIB and unphosphorylated eIF2 to eIF2B are likely independent.

Antagonism between eIF2(αP) and ISRIB Binding to eIF2B *In Vitro*

These structural insights predict mutually antagonistic binding of eIF2(αP) and ISRIB to eIF2B. To test this prediction, we measured the binding of a FAM-labeled ISRIB to eIF2B *in vitro* (Zyryanova et al., 2018). The binding of the small FAM-ISRIB (molecular weight [MW] ~1 kDa) to the much larger wild-type or ISR-insensitive mutants eIF2B(δ^{E310K}) and eIF2B(δ^{L314Q}) (MW ~500 kDa) results in a similar marked increase in the fluorescence polarization signal (Figure 3A).

Challenge of the eIF2B·FAM-ISRIB complex with eIF2(αP) resulted in a concentration-dependent decrease in the fluorescence polarization signal at steady state with an IC_{50} ~0.25 μM (Figures 3B and 3C). The Hill slope of the reaction 2.4 suggests

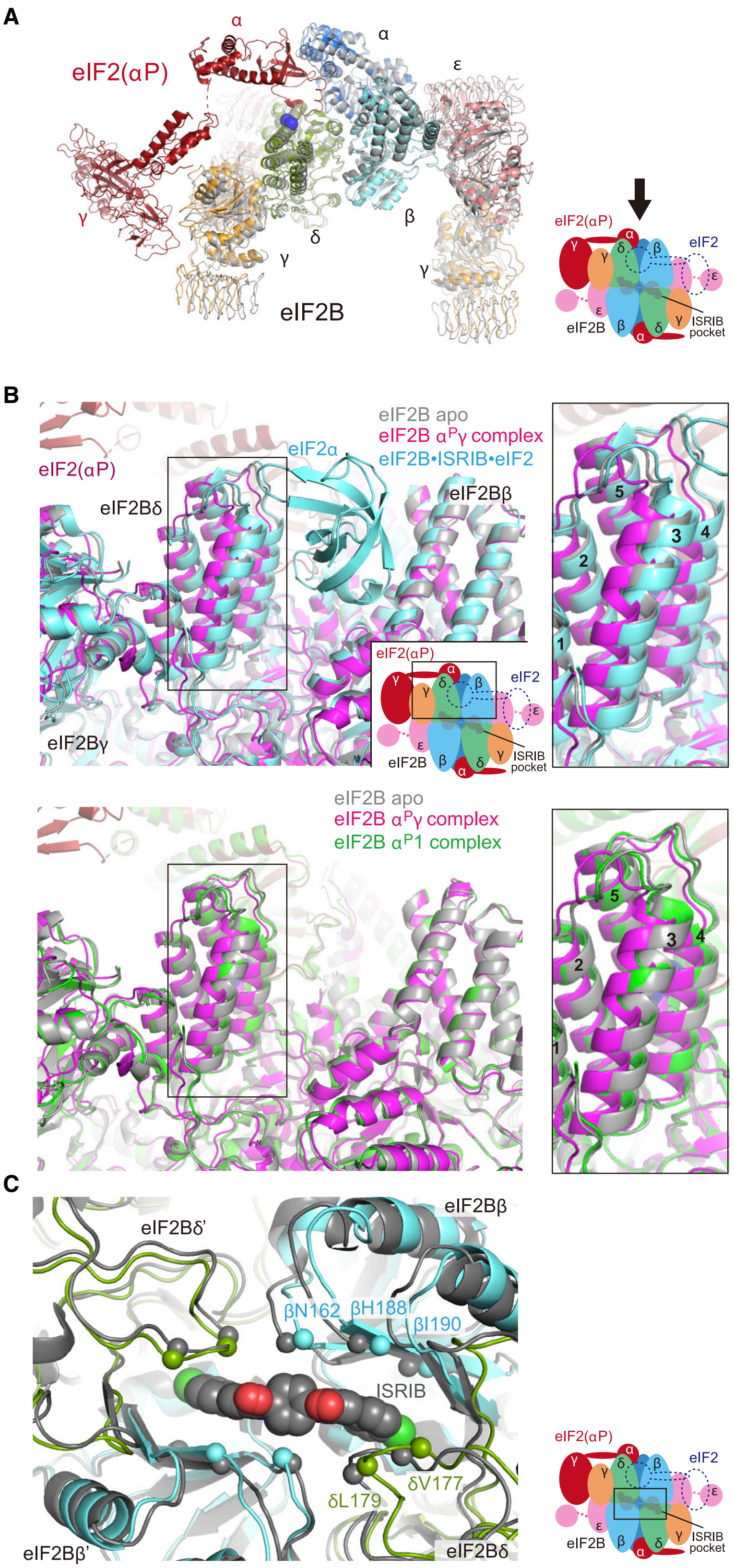

Figure 2. eIF2(αP) and ISRIB Associate with Different Conformations of eIF2B

(A) Overlay of the eIF2B apo structure (gray) and eIF2B in complex with two eIF2(αP) trimers (the $\alpha^P\gamma$ complex; color-coded as in the adjacent cartoon). The blue spheres show the position of the C^α atoms of eIF2Bδ^{E310} and δ^{L314}.

(B) Different arrangements of the eIF2B pocket that accommodates the eIF2α-NTD: unphosphorylated in the catalytically productive conformation, and phosphorylated in complexes containing one (α^P1) or two ($\alpha^P\gamma$) bound eIF2(αP) trimers. Upper panels: an overlay of the productive eIF2B·ISRIB·eIF2 complex (cyan, PDB: 6O81), the eIF2B apo structure (gray), and the $\alpha^P\gamma$ complex (magenta). For clarity, only the unphosphorylated eIF2α-NTD of the eIF2B·ISRIB·eIF2 complex is shown. Lower panels: similar alignment of the apo structure, the $\alpha^P\gamma$ structure, and the α^P1 structure (green). Right panels: close-up views showing the displacements of helix δ-α3 between the different complexes.

(C) Deformation of the ISRIB-binding pocket in eIF2B with two bound eIF2(αP) trimers (the $\alpha^P\gamma$ complex). The eIF2B·ISRIB complex structure (PDB: 6CAJ) is shown in gray and the $\alpha^P\gamma$ complex in color-coded representation (as in the adjacent cartoon). Key residues known to affect the binding or action of ISRIB are highlighted as spheres.

Structures are aligned by the four C-terminal domains of the β- and δ-subunits of eIF2B for (A) and (B), and by the C^α atoms surrounding (within 10 Å) the ISRIB molecule in the eIF2B·ISRIB structure for (C).

See also Figures S1 and S2.

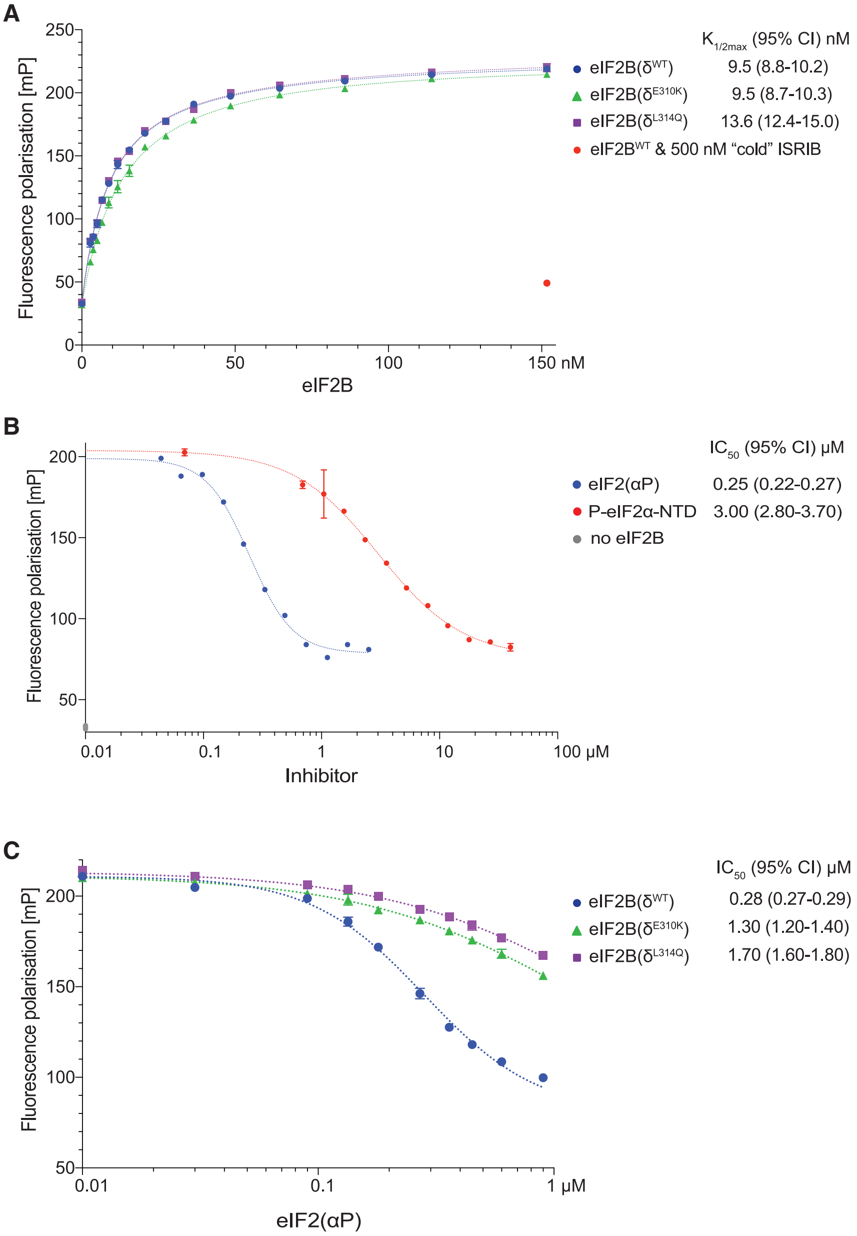

Figure 3. Phosphorylated eIF2 Attenuates FAM-ISRIB Binding to eIF2B

(A) Plot of fluorescence polarization signals (mean ± SD, n = 3) arising from samples of FAM-ISRIB (2.5 nM) incubated with varying concentrations of wild-type or mutant eIF2B. Where indicated, 500 nM unlabeled ISRIB was added as a competitor. $K_{1/2max}$ with 95% confidence intervals (CI) is shown.

(B) Plot of fluorescence polarization signals, at equilibrium, arising from FAM-ISRIB bound to wild-type eIF2B in presence of the indicated concentration of the P-eIF2α-NTD (mean ± SD, n = 3) or eIF2(αP) trimer. The data were fitted by non-linear regression analysis to a "log[inhibitor] versus response four parameter" model. IC_{50} values with 95% CI are shown.

(C) As in (B) above, plot of fluorescence polarization signals, at equilibrium, arising from FAM-ISRIB bound to wild-type or mutant eIF2B (100 nM) in presence of the indicated concentration of eIF2(αP) trimer (mean ± SD, n = 3). IC_{50} values with 95% CI are shown.

a cooperative process, consistent with enhanced displacement of FAM-ISRIB, when eIF2B is bound by two molecules of eIF2(αP). The isolated P-eIF2α-NTD also displaced FAM-ISRIB from eIF2B, but with an IC_{50} that was >10-fold higher (3.0 μM) (Figure 3B). Complexes formed between FAM-ISRIB and the ISR-insensitive eIF2B(δ^{E310K}) and eIF2B(δ^{L314Q}) were more resistant to the inhibitory effect of eIF2(αP) (Figure 3C), consistent with their diminished sensitivity to eIF2(αP) and their wild-type affinity for FAM-ISRIB (Figure 3A).

The eIF2B·FAM-ISRIB complex is maintained dynamically: unlabeled ISRIB displaced FAM-ISRIB from eIF2B with k_{off} of 0.74 min^{-1} (Figure 4A). The presence of eIF2 did not affect the stability of the eIF2B·ISRIB complex over time (Figure 4B, top). However, introduction of the PERK kinase into the assay (in presence of ATP), which resulted in the gradual phosphorylation of eIF2, led to a time-dependent loss of the fluorescence polarization signal (Figure 4B, top). The PERK-dependent decline in signal was enzyme concentration-dependent, it correlated with eIF2 phosphorylation (compare blue, lilac, and red square traces in Figure S3A) and recovered in a time-dependent manner by introducing phosphatases that dephosphorylated eIF2 (Figure S3B). These features attest to the dynamism and reversible nature of this *in vitro* representation of the ISR in the presence of ISRIB.

The time-dependent PERK-mediated loss of fluorescence polarization signal was not evident when wild-type eIF2 was replaced by a mutant eIF2(α^{S51A}) that is unable to serve as a substrate for PERK (Figure 4B, bottom). eIF2 phosphorylation-mediated loss of fluorescence polarization signal arising from FAM-ISRIB binding to wild-type eIF2B was attenuated by the ISR-insensitive mutants, eIF2Bδ^{E310K} and eIF2Bδ^{L314Q} (Figure 4B, top). These last findings confirm that the ability of eIF2(αP) to lower eIF2B's affinity for ISRIB *in vitro* is responsive to mutations that render eIF2B less sensitive to the ISR-inducing effects of eIF2(αP) in cells.

To assess the impact of ISRIB on the association of phosphorylated eIF2 with eIF2B, we turned to biolayer interferometry (BLI). The biotinylated P-eIF2α-NTD, immobilized via streptavidin to a BLI sensor, gave rise to a greater optical signal when reacted with fully assembled eIF2B decamers in solution compared to either eIF2Bβδγε tetramers (Figure 5A), or the ISR-insensitive mutants, eIF2B (δ^{E310K} or δ^{L314Q}) (Figure 5B). These features suggested that physiologically relevant contacts between eIF2B and the P-eIF2α-NTD contributed measurably to the BLI signal.

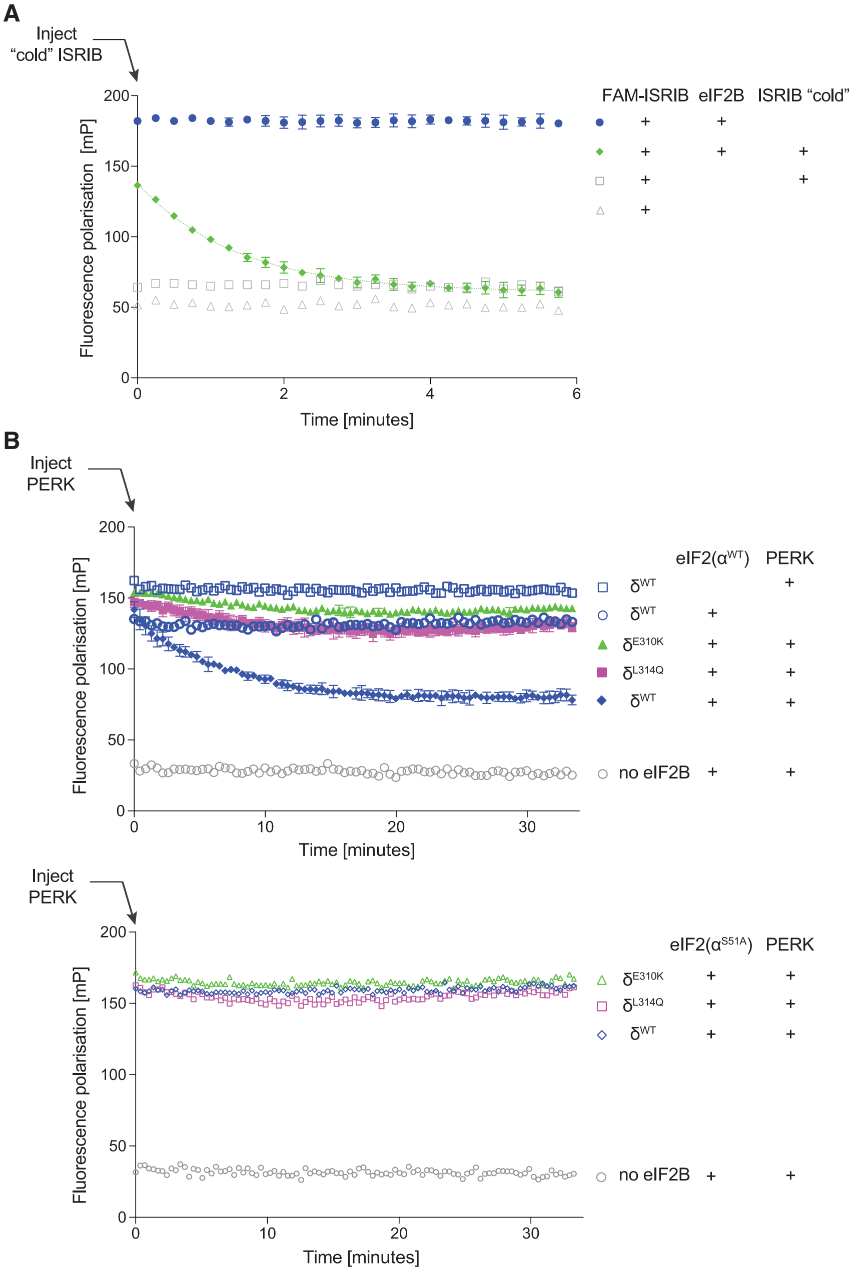

Figure 4. Phosphorylated eIF2 Attenuates FAM-ISRIB Binding to eIF2B on a Timescale Consistent with the ISR

(A) Plot of the time-dependent change in fluorescence polarization of FAM-ISRIB bound to wild-type eIF2B, following injection of 1 μM unlabeled (“cold”) ISRIB at t = 0 (green diamonds, mean ± SD, n = 3, and the fit of the first 6 min to a first order decay reaction [k_{off} = 0.74 min^{-1}; 95% CI, 0.68–0.9 min^{-1}; R^2, 0.9823], dotted green line). Control samples, unchallenged by “cold” ISRIB (blue circles, mean ± SD, n = 3) and reference samples (n = 1) from the same experiment are shown.

(B) Plot of time-dependent change in fluorescence polarization of FAM-ISRIB bound to wild-type or ISR-insensitive mutant eIF2Bs (δ^{E310K} or δ^{L314Q}) (60 nM) in presence or absence of 600 nM unphosphorylated wild-type eIF2 (top panel) or non-phosphorylatable eIF2(α^{S51A}) (bottom panel). Where indicated, at t = 0 the eIF2α kinase PERK was introduced to promote a pool of eIF2(αP). Shown are the mean ± SD (n = 3) of the fluorescence polarization values of the PERK-injected samples. The traces were fitted to a first order decay reaction. δ^{WT}: $t_{1/2}$ 4.7 min (95% CI 4.5–6.7, R^2, 0.9344); δ^{E310K}: $t_{1/2}$ 153 min, and δ^{L314Q}: $t_{1/2}$ 92 min (both with a poor fit to first order decay, R^2 <0.5).

See also Figure S3.

The presence of ISRIB attenuated the association of the P-eIF2α-NTD with eIF2B, across a range of eIF2B concentrations. The association and dissociation reactions detected by BLI were multi-phasic and, therefore, likely comprised of more than one binding event. Nonetheless, both the association phase and the dissociation phase gave a good fit to double exponential models. This enabled estimation of ISRIB’s effect on both eIF2B’s steady state binding ($K_{1/2max}$ of eIF2B-dependent BLI signal increased from 15.2 nM in the absence of ISRIB to 29.8 nM in its presence) (Figure 5B) and on the kinetics of eIF2B dissociation (ISRIB increased the PercentFast dissociation from 50% to 75%, with an EC_{50} of 1.8 nM) (Figure 5C). The difference between the $K_{1/2max}$ of eIF2B binding to the immobilized P-eIF2α-NTD in the BLI experiment and the IC_{50} of P-eIF2α-NTD’s inhibitory effect on FAM-ISRIB binding to eIF2B (Figure 3B) might reflect the occupancy of both regulatory sites of eIF2B in the maximally inhibited state in the later assay and the limitation of occupancy to a single site on eIF2B in the BLI experiment (Figure 5B). Also notable is the observation that the presence of the unphosphorylated eIF2α-NTD did not affect ISRIB’s ability to destabilize the P-eIF2α-NTD·eIF2B complex (Figure S4A). This finding is consistent with the lack of measurable cooperativity in the binding of ISRIB and unphosphorylated eIF2 to eIF2B (Figure S4B) and the equivalent structures of eIF2B when bound to eIF2 in presence or absence of ISRIB (Figures S2B and S2D).

Together, these experiments point to antagonism between engagement of eIF2(αP) and ISRIB as eIF2B ligands, at their respective distinct sites. Given that ISRIB binding to eIF2B favors, while eIF2(αP) binding disfavors, binding of unphosphorylated eIF2 as a substrate for nucleotide exchange, these findings suggest a plausible mechanism whereby ISRIB-mediated stabilization of the active conformation of the eIF2B decamer allosterically antagonizes the ISR.

Attenuated ISRIB Action in Cells Lacking eIF2(αP)

To learn more about the relative roles of allostery and eIF2B assembly in ISRIB’s action *in vivo*, we turned to cells lacking all phosphorylated eIF2. The ISR in CHO cells, in which the

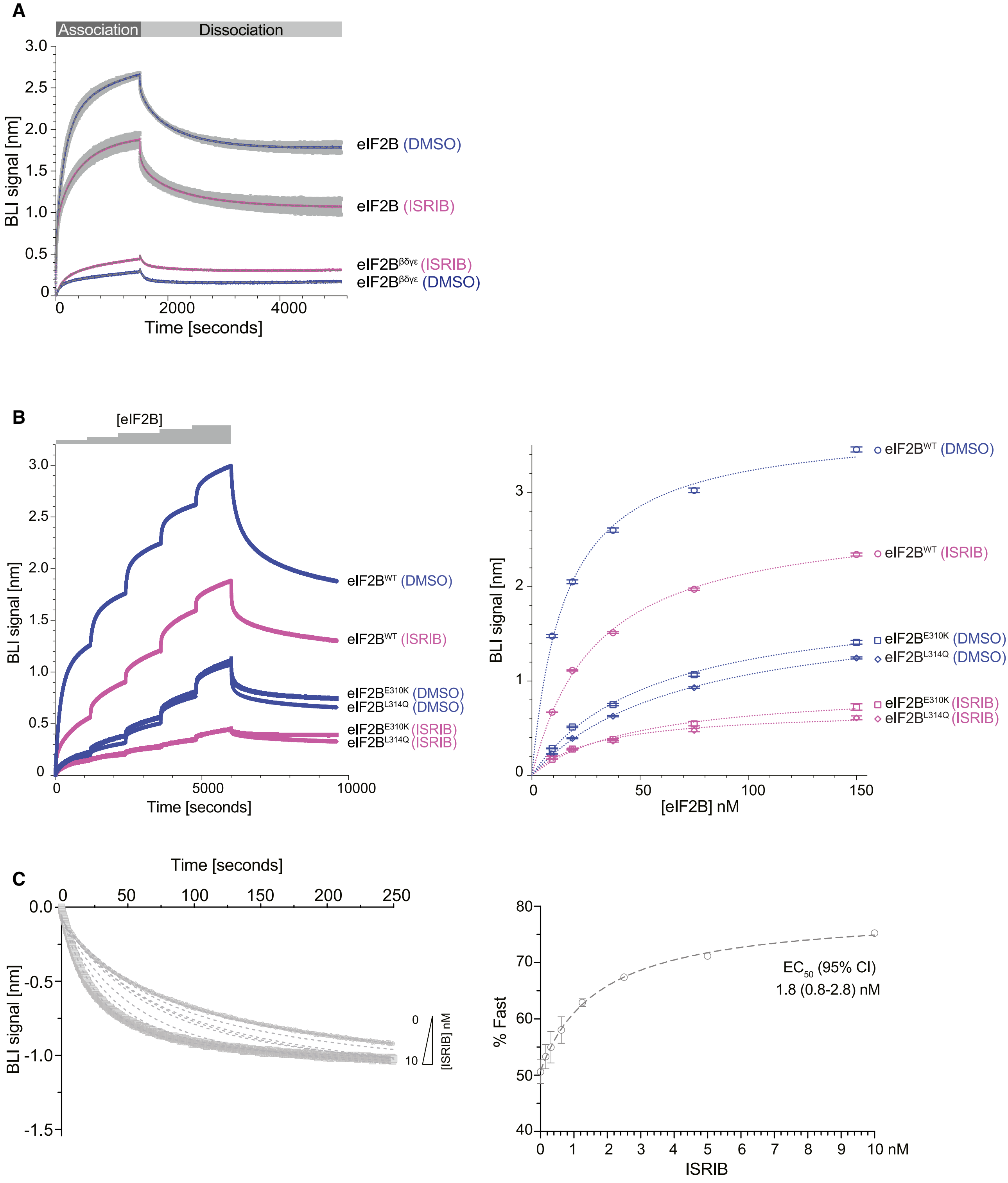

Figure 5. ISRIB Inhibits Binding of eIF2B to the N-Terminal Domain of Phosphorylated eIF2α

(A) Biolayer interferometry (BLI) traces of the association and dissociation phases of eIF2B decamers (100 nM) or eIF2B$^{\beta\delta\gamma\varepsilon}$ tetramers (400 nM) in the absence (DMSO, in blue) or presence of ISRIB (1 μM, in pink) to and from the biotinylated P-eIF2α-NTD immobilized on the BLI probe. The fits to a 2-phase association and a 2-phase dissociation model are indicated by the gray dashed line. Shown are mean ± SD (n = 3) of the sample of eIF2B decamers with and without ISRIB and all the data points of the tetramer samples from a representative experiment conducted three times.

(legend continued on next page)

wild-type eIF2α-encoding gene (*Eif2S1*) had been replaced by an *Eif2S1*^{S51A} mutant allele (encoding non-phosphorylatable eIF2α^{S51A}), is unresponsive to manipulations that activate eIF2α kinases (Crespillo-Casado et al., 2017) (Figure 1C). However, in these *Eif2S1*^{S51A} mutant cells, CRISPR/Cas9 disruption of eIF2B subunit-encoding genes (β, *Eif2b2*; δ, *Eif2b4*; ε, *Eif2b5*) activated the ISR, as reflected in the time-dependent emergence of a population of cells expressing high levels of CHOP::GFP (Figure 6A). Despite the progressive loss of viability following depletion of eIF2B (reflected in the decline in the CHOP::GFP-bright right-hand side subpopulation, observed 96 h after transduction with gene-specific guides and Cas9), this assay enabled the measurement of ISRIB's effect on the ISR in absence of any phosphorylated eIF2.

ISRIB had only a very modest (albeit statistically significant) inhibitory effect on the magnitude of the ISR induced by eIF2B subunit depletion, despite comparable levels of CHOP::GFP activation to those observed in L-histidinol-treated wild-type cells (Figure 6A, compare to Figure 1C). This finding—a weak ISRIB effect under conditions of eIF2B subunit depletion and no eIF2 phosphorylation—is consistent with the *in vitro* observation that, in the absence of eIF2(αP), ISRIB only weakly stimulated the nucleotide exchange activity of eIF2B, even when the enzyme's concentration was lowered by dilution (Figure 1A) (Tsai et al., 2018). Thus, it appears that while ISRIB's high-affinity binding to eIF2B can undoubtedly stabilize both the assembled decamer and its intermediates *in vitro* (Tsai et al., 2018), the contribution of this mechanism to its action in CHO cells is rather limited.

The aforementioned considerations are in keeping with the finding that density gradients of cell lysates do not support the existence of two substantial pools of eIF2B subunits: one of assembled decamers and another of unassembled eIF2B intermediates (Sidrauski et al., 2015; Zyryanova et al., 2018). Nonetheless, scrutiny of immunoblots of density gradients of both CHO and HeLa cell lysates (prepared under physiological salt conditions) does suggest a small but conspicuous pool of tagged endogenous eIF2Bγ (or eIF2Bβ) subunits migrating in the gradient at the position expected of an eIF2Bβδγε tetramer (MW 229 kDa). In both cell types, this minor pool of putative assembly intermediates appears to be depleted by ISRIB (Figure 6B). The latter observation is consistent with a measure of ISRIB-mediated acceleration of eIF2B's assembly and also suggests that the limited pool of unassembled intermediates may account for ISRIB's limited residual effect on the ISR, observed in *Eif2S1*^{S51A} mutant cells.

Depletion of eIF2B subunits, by interfering with their production (through CRISPR/Cas9-mediated gene disruption), is predicted to cut off the supply of even this modest pool of precursors and deprive ISRIB of an opportunity to increase eIF2B's activity via enhanced assembly. Therefore, to gauge the importance of accelerated assembly to ISRIB action in a different experimental system, we depleted cells of eIF2B's substrate by inactivating the eIF2α-encoding gene (*Eif2S1*) in the *Eif2S1*^{S51A} cells. As expected, this manipulation also activated the ISR, despite the absence of any phosphorylated eIF2α. However, in this scenario too, the stimulatory effect of ISRIB was very modest (compare Figure 6C with Figure 1C). Together, these findings suggest that in CHO cells, ISRIB reversal of the ISR is realized mostly through its ability to antagonize the effects of eIF2(αP) on pre-existing eIF2B decamers.

DISCUSSION

Comparing experimental systems containing and lacking phosphorylated eIF2 demonstrated the importance of eIF2(αP) to unveil ISRIB's ability to promote nucleotide exchange *in vitro* or ISR inhibition in cells. This correlates with structural observations whereby ISRIB binding is associated with a conformation of eIF2B conducive to binding of eIF2 as a substrate, while eIF2(αP) binding is associated with a different conformation of eIF2B with an altered ISRIB-binding pocket. Both an inhibitory effect of eIF2(αP) on ISRIB binding to eIF2B and a reciprocal inhibitory effect of ISRIB on the association between the P-eIF2α-NTD and eIF2B are observed *in vitro*. Together, these findings point to an allosteric component of ISRIB action, whereby its binding to eIF2B stabilizes the latter in a conformation that is relatively resistant to eIF2(αP). Given eIF2(αP)'s role as the major known upstream inducer of the ISR, this proposed allosteric mechanism goes some way to explaining ISRIB's ability to antagonize this cellular response to stress.

Both the dependence of ISRIB-mediated stimulation of eIF2B's nucleotide exchange activity on the presence of eIF2(αP) *in vitro* (Wong et al., 2018), and an apparent incompatibility between ISRIB binding to eIF2B and the conformation imposed on eIF2B by eIF2(αP) (Gordiyenko et al., 2019) had been suggested previously. Furthermore, while particles of ternary complexes of eIF2B·ISRIB·eIF2 (PDB: 6O81) are readily attainable (Kenner et al., 2019), efforts to assemble similar particles with eIF2B, ISRIB and eIF2(αP) have been unsuccessful. Our findings

(B) Left: BLI traces of consecutive association phases and a terminal dissociation phase of wild-type and the indicated eIF2B mutants in the absence (DMSO, in blue) or presence of ISRIB (1 μM, in pink) as in (A). The probe was reacted with escalating concentrations of eIF2B (9–150 nM) ± ISRIB, before dissociation in the respective buffer. Shown are all the data points of a representative experiment performed three times. Right: plots of the mean and 95% confidence interval (CI) of the plateau values of the association phases (obtained by fitting the data from the traces on the left to a 2-phase association non-linear regression model) against the concentration of eIF2B. The dotted line reports on the fit of plots to a one site specific binding with Hill slope = 1 non-linear regression model.

(C) Left: time-dependent change in BLI signal in the dissociation phase of eIF2B (previously associated in absence of ISRIB) from the biotinylated P-eIF2α-NTD in the presence of escalating concentration of ISRIB. The mean and 95% CI of the fraction of the dissociation attributed to the fast phase (%Fast) was calculated by fitting the dissociation traces to a biphasic model. The fit is indicated by the gray dotted lines. The overlaying data points in light gray are shown for 0 nM (small circles on top curve) and 10 nM (large squares on bottom curve) ISRIB only. Shown are traces from a representative experiment performed three times. Right: plot of the %Fast of the dissociation reactions to the left, as a function of ISRIB concentration. The plot was fitted to an [Agonist] versus response (Hill slope = 1) non-linear regression model (dotted line) yielding an EC_{50} of 1.8 (95% CI, 0.8–2.8) nM.

See also Figure S4.

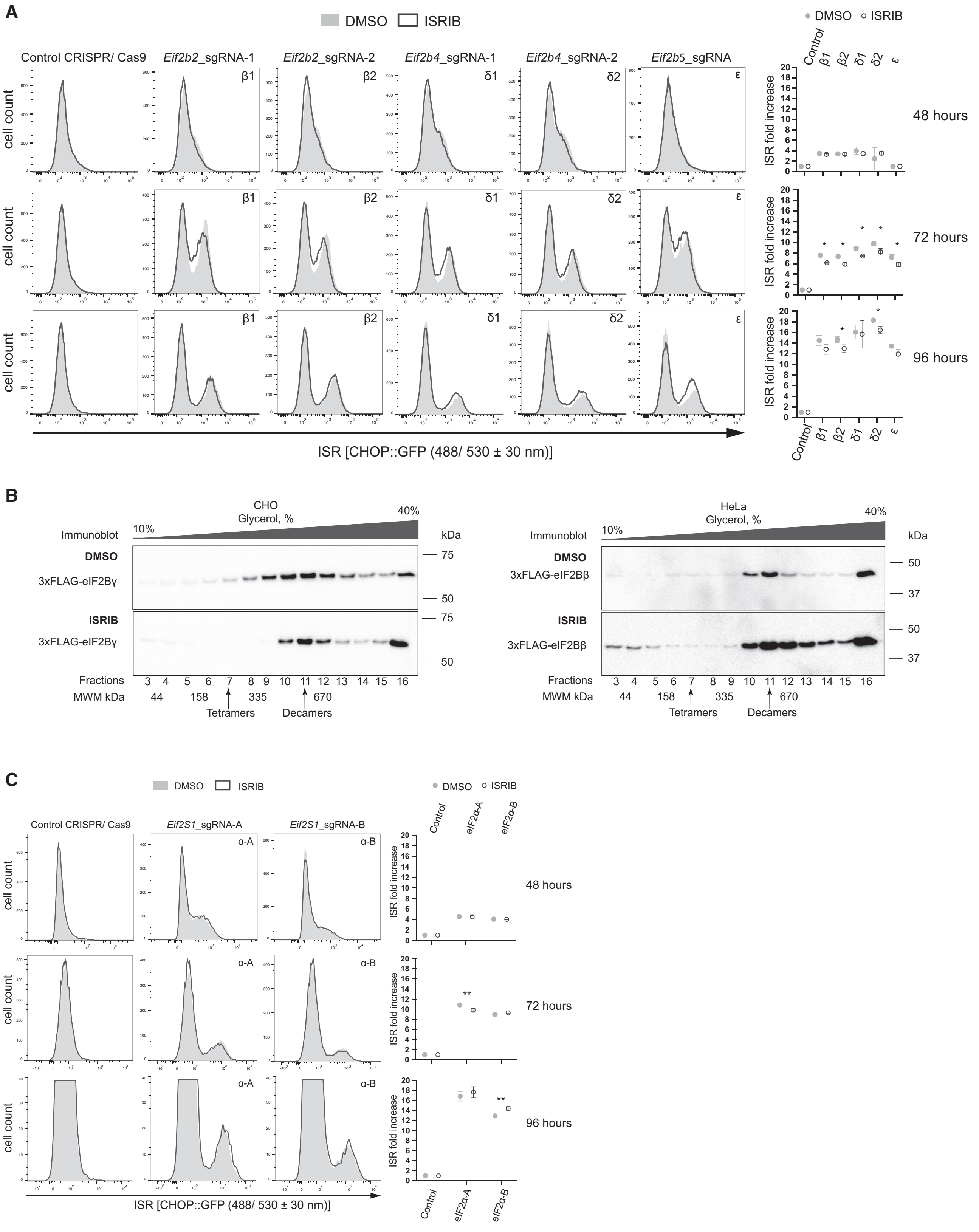

(legend on next page)

here unify these earlier clues, by highlighting the independent binding of ISRIB and unphosphorylated eIF2 to eIF2B and by supporting the conclusion that eIF2(αP) and ISRIB are incompatible ligands of eIF2B.

Structural analysis suggests at least two components to the aforementioned incompatibility. The first relates to changes imposed on the regulatory cleft of eIF2B by binding of the P-eIF2α-NTD between the α- and δ-subunits of eIF2B. Such changes appear to be enforced cooperatively by binding of two molecules of the P-eIF2α-NTD at both regulatory sites of eIF2B (Figure 7A). These contacts toggle eIF2B to a non-ISRIB binding mode, as demonstrated by the attenuated effect of eIF2(αP) on the binding of FAM-ISRIB to the ISR-desensitized eIF2B(δ^{E310K}) and eIF2B(δ^{L314Q}). The second relates to a role for the β- and γ-subunits of eIF2(αP), since the rearrangement of eIF2B was more prominent in structures containing the phosphorylated eIF2 trimer compared with those of eIF2B complexed with isolated P-eIF2α. This finding is mirrored in the ~10-fold lower IC_{50} of the eIF2(αP) trimer, compared with the isolated P-eIF2α-NTD, in the inhibition of FAM-ISRIB binding to eIF2B. It is tempting to speculate that contacts between the γ-subunit of eIF2(αP) and eIF2Bγ observed in some classes of particles in the cryo-EM images may stabilize the inhibited eIF2B·eIF2(αP) complex, but this issue has yet to be examined experimentally.

The β- and γ-subunits of phosphorylated eIF2, bound on one end of eIF2B, have previously been noted to block access of a second, unphosphorylated eIF2 (bound *in trans* to the regulatory domain on the opposite end of eIF2B) to eIF2B's catalytic site, on its bipartite ε-subunit (Kashiwagi et al., 2019) (Figure S2A). This mechanism favors partial inhibition of the catalytic activity of eIF2B when it accommodates a single eIF2(αP) trimer (state I in Figure 7B). Here, we note that the displacement of eIF2Bδ away from eIF2Bβ, observed in the eIF2B·eIF2(αP) complex with two bound eIF2(αP) trimers (the $\alpha^{P}2$ and $\alpha^{P}\gamma$ structures), widens the groove that could otherwise productively engage the eIF2α-NTD of a third unphosphorylated eIF2 trimer as a substrate, and is thus predicted to destabilize an active enzyme-substrate complex also *in cis* (on the same side as the bound eIF2(αP), as cartooned in Figure 7B state II). Higher concentrations of eIF2(αP) are likely to favor this strongly inhibited state.

Simultaneous binding of eIF2(αP) to both regulatory sites deforms the ISRIB-binding pocket. It is plausible that the rigid, largely helical structure of eIF2B's regulatory pocket (comprised of the helical NTDs of its α-, β-, and δ-subunits) (Kuhle et al., 2015) contributes to this allosteric coupling, rendering the concurrent binding of ISRIB with two molecules of eIF2(αP) unlikely. At low levels of eIF2(αP), the competition thus set up enables ISRIB to antagonize the transition of eIF2B from the fully active to the strongly inhibited state (the one most incompatible with ISRIB binding, Figure 7C) thereby dampening the cellular response to increasing levels of eIF2 phosphorylation. The kinetic parameters governing this antagonistic allostery have yet to be determined. However, the observation that ISRIB is only a partial antagonist of the ISR (Halliday et al., 2015; Rabouw et al., 2019) suggests that at high enough concentrations eIF2(αP) can outcompete ISRIB.

To parse the contribution of the allosteric antagonism between ISRIB and eIF2(αP) demonstrated here from the role of ISRIB in accelerating assembly of eIF2B decamers (Tsai et al., 2018), we experimentally activated the ISR in *Eif2S1*^{S51A} cells (bearing a non-phosphorylatable S51A mutation on eIF2 α-subunit and thus lacking any P-eIF2α) by transient genetic manipulations that deplete their pool of ternary eIF2·GTP·Met-$tRNA_i$ complexes. In absence of eIF2(αP), the limited velocity of the nucleotide exchange reaction, imposed by either substrate or enzyme depletion, resulted in an ISR that was only weakly antagonized by ISRIB. These findings argue against an important role in ISRIB action for accelerated assembly or stabilization of eIF2B in CHO cells. This conclusion also fits with the paucity of evidence for a substantial pool of eIF2B precursors for ISRIB to draw on and accelerate assembly of eIF2B in cells under basal conditions. Nor is there evidence to suggest that the eIF2B decamer is in a rapid exchange equilibrium containing a significant fraction of eIF2Bβδγε tetramers and eIF2Bα_2 dimers, as such an equilibrium would be expected to be skewed by ISRIB toward the active GEF eIF2B decamer *in vitro* and *in vivo*, even in absence of eIF2(αP).

Limitations of Study

It is noteworthy that we have not ruled out the possibility that eIF2(αP) may itself perturb the decamer-tetramer equilibrium of eIF2B, thus uncovering the potential stabilizing activity of ISRIB as a basis for ISR inhibition. However, efforts to otherwise favor the dissolution of decamers in the absence of eIF2(αP), by dilution to a concentration 20- to 100-fold lower than that found in cells (53–293 nM, Hein et al., 2015) (Figure 1A) or by *in vivo*

Figure 6. Attenuated ISRIB Action in Cells Lacking eIF2(αP)

(A) Characterization of the ISR in *Eif2S1*^{S51A} mutant CHO cells (lacking phosphorylatable eIF2α) depleted of eIF2B subunits by CRISPR/Cas9 targeting of their encoding genes. Two different guides for either beta (β1, β2) or delta (δ1, δ2) subunit, and one guide for epsilon subunit (ε) were transfected separately. Where indicated, the cells were exposed continuously to ISRIB (1 μM), commencing at the point of transduction with the CRISPR/Cas9 encoding plasmids and continued until harvest. Shown are histograms of the CHOP::GFP ISR reporter in populations of cells 48, 72, and 96 h following eIF2B gene targeting (±ISRIB) from a representative experiment performed three times. The mean ± SD (n = 3) of the ratio of fluorescent signal of the ISR-induced population (the right peak on the histograms) to the non-induced (left peak) are plotted to the right (*p < 0.05 Student's t test).

(B) Immunoblot of 3xFLAG-tagged endogenous eIF2Bγ detected with anti-FLAG M2 antibodies in CHO or 3xFLAG-tagged endogenous eIF2Bβ in HeLa cell lysates that were either treated with DMSO (top panel) or ISRIB (bottom panel) and resolved on a 10%–40% glycerol density gradient in a buffer of physiological salt concentration. The position of reference proteins of the indicated molecular weight in this gradient is indicated below the image and the arrows point to the predicted position of eIF2Bβδγε tetramers and eIF2B$(\alpha)_2(\beta\delta\gamma\varepsilon)_2$ decamers.

(C) As in (A) above, but following CRISPR/Cas9-mediated depletion of eIF2B's substrate eIF2, by targeting the *Eif2S1* gene encoding its α-subunit. Two different guides, eIF2α-A and eIF2α-B, were transfected separately. Shown is a representative experiment performed twice. The mean ± SD (n = 2) of fluorescent signals ratio as in (A) are plotted to the right (**p < 0.05 Student's t test).

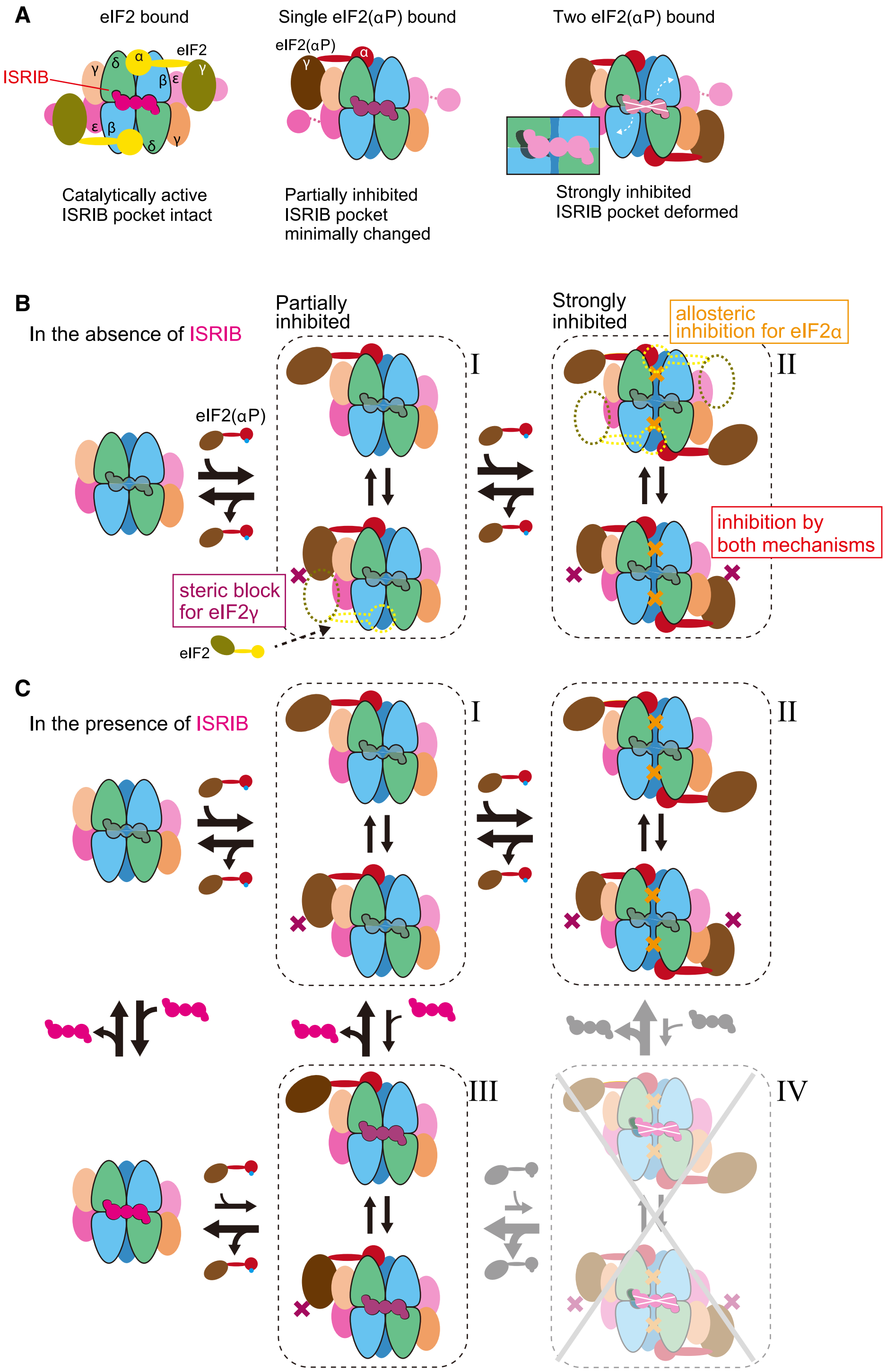

Figure 7. A Model of the Functional Consequences of the Antagonism between eIF2(αP) and ISRIB Binding to eIF2B

(A) Cartoon of the ISRIB-binding pocket in the active (ground) state of eIF2B (left), eIF2B bound by one eIF2(αP) trimer (center), and eIF2B bound by two eIF2(αP) trimers (right).

(B) The binding of one eIF2(αP) trimer partially inhibits the catalytic activity by a steric block of the active site induced by the docking of the γ-subunit of eIF2(αP) onto eIF2Bγ (state I). Binding of a second eIF2(αP) trimer occludes the second active site of eIF2B by a similar steric block but also interferes with catalytic activity through allosteric inhibition that deforms both pockets for productive binding of eIF2α, strongly inhibiting the catalytic activity of eIF2B (state II).

(legend continued on next page)

CellPress
OPEN ACCESS

depletion of individual eIF2B subunits (Figure 6A), did not result in the manifestation of marked ISRIB effects. A different scenario may arise if eIF2Bα becomes limiting. The residual guanine nucleotide exchange activity of eIF2B(βδγε)$_2$ octamers would rise to prominence, potentially unveiling a role for their ISRIB-mediated stabilization (Tsai et al., 2018) that could operate independently of eIF2(αP) and contribute to the ISR antagonism observed in ISRIB-treated *Eif2S1*^{S51A} cells.

The relative contribution of allostery and stabilization to ISRIB's action may be different in cells with mutations in eIF2B that lower its enzymatic activity by destabilizing the decamer. Stabilization may therefore contribute to the salubrious role of ISRIB (and the related compound 2BAct) in cellular and animal models of the myelinopathy associated with eIF2B mutations (Wong et al., 2018, 2019; Abbink et al., 2019). It is also possible that stabilization of the eIF2B decamer may be important in other contexts, such as regulation of eIF2B by subunit phosphorylation (Wang et al., 2001) or in other cell types that may have significant unassembled pools of eIF2B precursors (Hodgson et al., 2019)

Given the discovery of ISRIB's role as an allosteric regulator of eIF2B presented here, it is interesting to contemplate the potential contribution of decamer assembly/stability and allostery to the action of other ligands of the ISRIB pocket—be they yet-to-be discovered physiological regulators of translation or drugs. Particularly interesting is the question of whether ligands of the ISRIB pocket can be discovered that stabilize the inactive conformation of eIF2B—the one imposed on it by eIF2(αP). If ISRIB attains its effects in cells largely by allostery, acting on a cellular pool of stable eIF2B decamers (as our findings here suggest), such anti-ISRIB compounds are predicted to increase eIF2B's affinity for eIF2(αP) and thus extend the ISR, which may be of benefit in some contexts (for example as anti-viral agents). Given that, like ISRIB, a subset of such ligands may also accelerate the assembly of the eIF2B decamer (at least *in vitro*), their activity as ISR modulators may shed light on the relative role of these two known facets of ISRIB action in cells.

STAR★METHODS

Detailed methods are provided in the online version of this paper and include the following:

- KEY RESOURCES TABLE
- RESOURCE AVAILABILITY
  - Lead Contact
  - Materials Availability
  - Data and Code Availability
- EXPERIMENTAL MODEL AND SUBJECT DETAILS
  - T7 Express competent *E. coli* strain
  - FreeStyle 293-F cells
  - T7 Express lysY/Iq competent *E. coli* strain
  - CHO-K1-derived adherent cell lines
  - HeLa-derived adherent cell lines
- METHOD DETAILS
  - Protein preparation
  - Guanine nucleotide exchange activity
  - Cryo-EM analysis
  - ISRIB binding to eIF2B
  - eIF2B binding to phosphorylated eIF2α
  - Measurement of the ISR in cultured cells
  - eIF2B and eIF2 subunits depletion
  - Introduction of ISR resistant mutations into cultured cells
  - Glycerol gradient fractionation of cell lysates
- QUANTIFICATION AND STATISTICAL ANALYSIS

SUPPLEMENTAL INFORMATION

Supplemental Information can be found online at https://doi.org/10.1016/j.molcel.2020.10.031.

ACKNOWLEDGMENTS

We thank Yahui Yan for purifying recombinant eIF2α-NTD, Steffen Preissler and Lisa Neidhardt (CIMR) for critiques of the manuscript, Alan Hinnebusch and Tom Dever (NIH/NICHD) for suggesting the experiment shown in Figure 6C, Reiner Schulte, Chiara Cossetti, and Gabriela Grondys-Kotarba (CIMR core flow cytometry facility) for their help and advice on cell sorting, and Akeel Abu Alard and Peter Fischer (U Nottingham) for the gift of ISRIB and FAM-ISRIB. This work was supported by a Wellcome Trust Principal Research Fellowship to D.R. (Wellcome 200848/Z/16/Z), Japan Society for the Promotion of Science (Grant-in-Aid for Scientific Research [B], JP19H03172 to T.I. and Grants-in-Aid for Early-Career Scientists JP18K14644 to K.K.), Japan Agency for Medical Research and Development (JP20am0101115, JP20am0101082, and JP20gm1410001), and RIKEN (the Integrated Life Science Research to Challenge Super Aging Society and the Pioneering Project "Dynamic Structural Biology").

AUTHOR CONTRIBUTIONS

A.F.Z., K.K., T.I., and D.R. conceived the study, designed experiments, analyzed data, prepared figures and tables, and co-wrote the manuscript. K.K., A.S., M.N., M.Y., M.S., A.F.Z., D.R., and L.A.P. purified recombinant proteins. K.K., M.S., and T.I. conducted cryo-EM structural work. A.F.Z. and D.R. conducted GEF, FP, and BLI assays. L.A.P. assisted in the design and interpretation of the BLI experiments. A.F.Z. and H.P.H. conducted analytical gradient centrifugation of cell lysates. A.F.Z., A.C.-C., and C.R. created and evaluated mutant cell lines. A.F.Z. and C.R. conducted CRISPR/Cas9-mediated depletion experiments in cells. All authors contributed to discussion and revision of the manuscript.

DECLARATION OF INTERESTS

The authors declare no competing interests.

Received: June 30, 2020
Revised: October 20, 2020
Accepted: October 22, 2020
Published: November 20, 2020

(C) The presence of ISRIB hierarchically antagonizes the binding of eIF2(αP). Weaker antagonism toward one bound eIF2(αP) trimer (states I and III), as ISRIB's binding pocket is less deformed under these circumstances. The stronger deformation of the ISRIB-binding pocket that is coupled to the binding of a second eIF2(αP) trimer sets up a competition whereby state IV is most strongly disfavored by ISRIB (rendered opaque in the cartoon). By stabilizing the ground state and the partially inhibited state III, ISRIB pulls the equilibrium away from the strongly inhibited state II, thus antagonizing the ISR. High enough concentrations of eIF2(αP) competitively override this inhibition.

REFERENCES

Abbink, T.E.M., Wisse, L.E., Jaku, E., Thiecke, M.J., Voltolini-González, D., Fritsen, H., Bobeldijk, S., Ter Braak, T.J., Polder, E., Postma, N.L., et al. (2019). Vanishing white matter: deregulated integrated stress response as therapy target. Ann. Clin. Transl. Neurol. *6*, 1407–1422.

Adams, P.D., Afonine, P.V., Bunkóczi, G., Chen, V.B., Davis, I.W., Echols, N., Headd, J.J., Hung, L.W., Kapral, G.J., Grosse-Kunstleve, R.W., et al. (2010). PHENIX: a comprehensive Python-based system for macromolecular structure solution. Acta Crystallogr. D Biol. Crystallogr. *66*, 213–221.

Adomavicius, T., Guaita, M., Zhou, Y., Jennings, M.D., Latif, Z., Roseman, A.M., and Pavitt, G.D. (2019). The structural basis of translational control by eIF2 phosphorylation. Nat. Commun. *10*, 2136.

Chou, A., Krukowski, K., Jopson, T., Zhu, P.J., Costa-Mattioli, M., Walter, P., and Rosi, S. (2017). Inhibition of the integrated stress response reverses cognitive deficits after traumatic brain injury. Proc. Natl. Acad. Sci. USA *114*, E6420–E6426.

Crespillo-Casado, A., Chambers, J.E., Fischer, P.M., Marciniak, S.J., and Ron, D. (2017). PPP1R15A-mediated dephosphorylation of eIF2α is unaffected by Sephin1 or Guanabenz. eLife *6*, e26109.

Crespillo-Casado, A., Claes, Z., Choy, M.S., Peti, W., Bollen, M., and Ron, D. (2018). A Sephin1-insensitive tripartite holophosphatase dephosphorylates translation initiation factor 2α. J. Biol. Chem. *293*, 7766–7776.

de Haro, C., Méndez, R., and Santoyo, J. (1996). The eIF-2alpha kinases and the control of protein synthesis. FASEB J. *10*, 1378–1387.

Dev, K., Qiu, H., Dong, J., Zhang, F., Barthlme, D., and Hinnebusch, A.G. (2010). The beta/Gcd7 subunit of eukaryotic translation initiation factor 2B (eIF2B), a guanine nucleotide exchange factor, is crucial for binding eIF2 in vivo. Mol. Cell. Biol. *30*, 5218–5233.

Emsley, P., Lohkamp, B., Scott, W.G., and Cowtan, K. (2010). Features and development of Coot. Acta Crystallogr. D Biol. Crystallogr. *66*, 486–501.

Gordiyenko, Y., Llácer, J.L., and Ramakrishnan, V. (2019). Structural basis for the inhibition of translation through eIF2α phosphorylation. Nat. Commun. *10*, 2640.

Halliday, M., Radford, H., Sekine, Y., Moreno, J., Verity, N., le Quesne, J., Ortori, C.A., Barrett, D.A., Fromont, C., Fischer, P.M., et al. (2015). Partial restoration of protein synthesis rates by the small molecule ISRIB prevents neurodegeneration without pancreatic toxicity. Cell Death Dis. *6*, e1672.

Harding, H.P., Zhang, Y., Zeng, H., Novoa, I., Lu, P.D., Calfon, M., Sadri, N., Yun, C., Popko, B., Paules, R., et al. (2003). An integrated stress response regulates amino acid metabolism and resistance to oxidative stress. Mol. Cell *11*, 619–633.

Hein, M.Y., Hubner, N.C., Poser, I., Cox, J., Nagaraj, N., Toyoda, Y., Gak, I.A., Weisswange, I., Mansfeld, J., Buchholz, F., et al. (2015). A human interactome in three quantitative dimensions organized by stoichiometries and abundances. Cell *163*, 712–723.

Hodgson, R.E., Varanda, B.A., Ashe, M.P., Allen, K.E., and Campbell, S.G. (2019). Cellular eIF2B subunit localization: implications for the integrated stress response and its control by small molecule drugs. Mol. Biol. Cell *30*, 942–958.

Kashiwagi, K., Takahashi, M., Nishimoto, M., Hiyama, T.B., Higo, T., Umehara, T., Sakamoto, K., Ito, T., and Yokoyama, S. (2016). Crystal structure of eukaryotic translation initiation factor 2B. Nature *531*, 122–125.

Kashiwagi, K., Yokoyama, T., Nishimoto, M., Takahashi, M., Sakamoto, A., Yonemochi, M., Shirouzu, M., and Ito, T. (2019). Structural basis for eIF2B inhibition in integrated stress response. Science *364*, 495–499.

Kenner, L.R., Anand, A.A., Nguyen, H.C., Myasnikov, A.G., Klose, C.J., McGeever, L.A., Tsai, J.C., Miller-Vedam, L.E., Walter, P., and Frost, A. (2019). eIF2B-catalyzed nucleotide exchange and phosphoregulation by the integrated stress response. Science *364*, 491–495.

Kimball, S.R., Fabian, J.R., Pavitt, G.D., Hinnebusch, A.G., and Jefferson, L.S. (1998). Regulation of guanine nucleotide exchange through phosphorylation of eukaryotic initiation factor eIF2alpha. Role of the alpha- and delta-subunits of eiF2b. J. Biol. Chem. *273*, 12841–12845.

Kuhle, B., Eulig, N.K., and Ficner, R. (2015). Architecture of the eIF2B regulatory subcomplex and its implications for the regulation of guanine nucleotide exchange on eIF2. Nucleic Acids Res. *43*, 9994–10014.

Pakos-Zebrucka, K., Koryga, I., Mnich, K., Ljujic, M., Samali, A., and Gorman, A.M. (2016). The integrated stress response. EMBO Rep. *17*, 1374–1395.

Pavitt, G.D., Yang, W., and Hinnebusch, A.G. (1997). Homologous segments in three subunits of the guanine nucleotide exchange factor eIF2B mediate translational regulation by phosphorylation of eIF2. Mol. Cell. Biol. *17*, 1298–1313.

Rabouw, H.H., Langereis, M.A., Anand, A.A., Visser, L.J., de Groot, R.J., Walter, P., and van Kuppeveld, F.J.M. (2019). Small molecule ISRIB suppresses the integrated stress response within a defined window of activation. Proc. Natl. Acad. Sci. USA *116*, 2097–2102.

Ranu, R.S., and London, I.M. (1979). Regulation of protein synthesis in rabbit reticulocyte lysates: additional initiation factor required for formation of ternary complex (eIF-2.GTP.Met-tRNAf) and demonstration of inhibitory effect of heme-regulated protein kinase. Proc. Natl. Acad. Sci. USA *76*, 1079–1083.

Schindelin, J., Arganda-Carreras, I., Frise, E., Kaynig, V., Longair, M., Pietzsch, T., Preibisch, S., Rueden, C., Saalfeld, S., Schmid, B., et al. (2012). Fiji: an open-source platform for biological-image analysis. Nat. Methods *9*, 676–682.

Schmidt, E.K., Clavarino, G., Ceppi, M., and Pierre, P. (2009). SUnSET, a nonradioactive method to monitor protein synthesis. Nat. Methods *6*, 275–277.

Scorsone, K.A., Panniers, R., Rowlands, A.G., and Henshaw, E.C. (1987). Phosphorylation of eukaryotic initiation factor 2 during physiological stresses which affect protein synthesis. J. Biol. Chem. *262*, 14538–14543.

Sekine, Y., Zyryanova, A., Crespillo-Casado, A., Fischer, P.M., Harding, H.P., and Ron, D. (2015). Stress responses. Mutations in a translation initiation factor identify the target of a memory-enhancing compound. Science *348*, 1027–1030.

Sekine, Y., Zyryanova, A., Crespillo-Casado, A., Amin-Wetzel, N., Harding, H.P., and Ron, D. (2016). Paradoxical Sensitivity to an Integrated Stress Response Blocking Mutation in Vanishing White Matter Cells. PLoS ONE *11*, e0166278.

Sidrauski, C., Acosta-Alvear, D., Khoutorsky, A., Vedantham, P., Hearn, B.R., Li, H., Gamache, K., Gallagher, C.M., Ang, K.K., Wilson, C., et al. (2013). Pharmacological brake-release of mRNA translation enhances cognitive memory. eLife *2*, e00498.

Sidrauski, C., Tsai, J.C., Kampmann, M., Hearn, B.R., Vedantham, P., Jaishankar, P., Sokabe, M., Mendez, A.S., Newton, B.W., Tang, E.L., et al. (2015). Pharmacological dimerization and activation of the exchange factor eIF2B antagonizes the integrated stress response. eLife *4*, e07314.

Tang, G., Peng, L., Baldwin, P.R., Mann, D.S., Jiang, W., Rees, I., and Ludtke, S.J. (2007). EMAN2: an extensible image processing suite for electron microscopy. J. Struct. Biol. *157*, 38–46.

Tsai, J.C., Miller-Vedam, L.E., Anand, A.A., Jaishankar, P., Nguyen, H.C., Renslo, A.R., Frost, A., and Walter, P. (2018). Structure of the nucleotide exchange factor eIF2B reveals mechanism of memory-enhancing molecule. Science *359*, eaaq0939.

Wang, X., Paulin, F.E., Campbell, L.E., Gomez, E., O'Brien, K., Morrice, N., and Proud, C.G. (2001). Eukaryotic initiation factor 2B: identification of multiple phosphorylation sites in the epsilon-subunit and their functions in vivo. EMBO J. *20*, 4349–4359.

Wong, Y.L., LeBon, L., Edalji, R., Lim, H.B., Sun, C., and Sidrauski, C. (2018). The small molecule ISRIB rescues the stability and activity of Vanishing White Matter Disease eIF2B mutant complexes. eLife *7*, e32733.

Wong, Y.L., LeBon, L., Basso, A.M., Kohlhaas, K.L., Nikkel, A.L., Robb, H.M., Donnelly-Roberts, D.L., Prakash, J., Swensen, A.M., Rubinstein, N.D., et al. (2019). eIF2B activator prevents neurological defects caused by a chronic integrated stress response. eLife *8*, e42940.

Zhang, K. (2016). Gctf: Real-time CTF determination and correction. J. Struct. Biol. *193*, 1–12.

CellPress
OPEN ACCESS

Zheng, S.Q., Palovcak, E., Armache, J.P., Verba, K.A., Cheng, Y., and Agard, D.A. (2017). MotionCor2: anisotropic correction of beam-induced motion for improved cryo-electron microscopy. Nat. Methods *14*, 331–332.

Zhu, P.J., Khatiwada, S., Cui, Y., Reineke, L.C., Dooling, S.W., Kim, J.J., Li, W., Walter, P., and Costa-Mattioli, M. (2019). Activation of the ISR mediates the behavioral and neurophysiological abnormalities in Down syndrome. Science *366*, 843–849.

Zivanov, J., Nakane, T., Forsberg, B.O., Kimanius, D., Hagen, W.J., Lindahl, E., and Scheres, S.H. (2018). New tools for automated high-resolution cryo-EM structure determination in RELION-3. eLife *7*, e42166.

Zyryanova, A.F., Weis, F., Faille, A., Alard, A.A., Crespillo-Casado, A., Sekine, Y., Harding, H.P., Allen, F., Parts, L., Fromont, C., et al. (2018). Binding of ISRIB reveals a regulatory site in the nucleotide exchange factor eIF2B. Science *359*, 1533–1536.

STAR★METHODS

KEY RESOURCES TABLE

REAGENT or RESOURCE	SOURCE	IDENTIFIER
**Antibodies**		
Mouse monoclonal anti-FLAG M2	Sigma Aldrich	RRID:AB_262044
Mouse monoclonal anti-puromycin	Schmidt et al., 2009	RRID:AB_2566826
Rabbit monoclonal anti-phospho-eIF2alpha-Ser51	AbCam	RRID: AB_732117
Mouse monoclonal anti-eIF2alpha (total)	Scorsone et al., 1987	N/A
**Bacterial and Virus Strains**		
T7 Express Competent *E. coli*	New England Biolabs	C2566I
T7 Express lysY/Iq competent *E. coli*	New England Biolabs	C3013
**Chemicals, Peptides, and Recombinant Proteins**		
trans-ISRIB	Sekine et al., 2015	Gift of Peter Fischer U. Nottingham
FAM-ISRIB	Zyryanova et al., 2018	Gift of Peter Fischer U. Nottingham
Human eIF2 trimer	Kashiwagi et al., 2019 and this study	N/A
Human eIF2B decamer	Kashiwagi et al., 2019 and this study	N/A
Human eIF2α-NTD	This study	N/A
BODIPY-FL-GDP	Invitrogen	G22360
**Deposited Data**		
Cryo-EM map of the α^P1 complex	This study	EMDB: EMD-30570
Cryo-EM map of the α^P2 complex	This study	EMDB: EMD-30569
Cryo-EM map of the $\alpha^P\gamma$ complex	This study	EMDB: EMD-30568
Cryo-EM map of eIF2B apo	This study	EMDB: EMD-30571
Coordinates of the α^P1 complex	This study	PDB: 7D45
Coordinates of the α^P2 complex	This study	PDB: 7D44
Coordinates of the $\alpha^P\gamma$ complex	This study	PDB: 7D43
Coordinates of the eIF2B apo structure	This study	PDB: 7D46
**Experimental Models: Cell Lines**		
CHO-S21 dual reporter [CHOP::GFP; Xbp1::Turquoise]	Sekine et al., 2016	N/A
CHO-S51A dual reporter ISR-insensitive eIF2α^{S51A}	Sekine et al., 2016	N/A
CHO-S21 [*Eif2b4*_L316N] ISR-insensitive	This study	N/A
CHO-S21 [*Eif2b4*_E312K; L316V] ISR-insensitive	This study	N/A
CHO-C30 CHOP::GFP-reporter eIF2Bγ-3xFlag-tagged	Zyryanova et al., 2018	N/A
HeLa [3 X Flag-*EIF2B2*]	Sekine et al., 2015	N/A
FreeStyle 293-F cells	Thermo Fisher Scientific	R79007
For full list see Table S1	N/A	N/A
**Oligonucleotides**		
Oligo2213_CHO_eIF2B4_Exon10_ssODN_L310X: GGTTTTTCAGTCAGGTATTCACCATACCATCCAT ATACCAGGATCACGTCCCCGTCACTGATCTTC TTAGAGGCAAACCGTGAAATTGCTTGAGCTGCN NNCACAATCTTCTCTTGTACATACCGATCAATG GCTTCTCTAAGTTCTGACTTTGCCTAAATGTTG AGAGAACAGTGATATAATTCACCC	Integrated DNA Technologies	N/A
Oligo2214_CHO_eIF2B4_Exon10_ssODN_E306K_L310X: GGTTTTTCAGTCAGGTATTCACCATACCATCCATATACC AGGATCACGTCCCCGTCACTGATCTTCTTAGAGGCAAA CCGTGAAATTGCTTGAGCTGCNNNCACAATCTTCTTTT GTACATACCGATCAATGGCTTCTCTAAGTTCTGACTTT GCCTAAATGTTGAGAGAACAGTGATATAATTCACCC	Integrated DNA Technologies	N/A

(*Continued on next page*)

Continued

REAGENT or RESOURCE	SOURCE	IDENTIFIER
For full list see Table S3	This study	N/A
Recombinant DNA		
UK2731_heIF2a_2-187_pSUMO3	This study	N/A
UK2733_heIF2a_2-187_WT_AviTag_H6_pET-30a(+)	This study	N/A
pETDuet-2B4-2B2_dE310K	This study	N/A
pETDuet-2B4-2B2_dL314Q	This study	N/A
pEBMulti-Neo-human-eIF2alpha-PA	This study	N/A
pEBMulti-Neo-human-eIF2alpha-S52A-PA	This study	N/A
pEBMulti-Neo	Fuji Film Wako	057-08131
For full list see Table S2	This study	N/A
Software and Algorithms		
GraphPad Prism version 8	https://www.graphpad.com:443/	N/A
Fiji (ImageJ 1.53c NIH)	Schindelin et al., 2012	N/A
RELION 3.0	Zivanov et al., 2018	N/A
Gautomatch	https://www.mrc-lmb.cam.ac.uk/kzhang/	N/A
EMAN2	Tang et al., 2007	N/A
Coot	Emsley et al., 2010	N/A
PHENIX	Adams et al., 2010	N/A
FlowJo	https://www.flowjo.com	N/A

RESOURCE AVAILABILITY

Lead Contact

Further information and requests for resources and reagents should be directed to and will be fulfilled by the Lead Contact, David Ron (dr360@medschl.cam.ac.uk).

Materials Availability

Plasmids and cell lines generated in this study are available upon written request to the Lead Contact. Please consult the list of unique reagents in Tables S1–S3 and Key Resources Table.

Data and Code Availability

The cryo-EM maps generated in this study are available at EMDB, entry EMD-30568 (the $\alpha^P\gamma$ complex), EMD-30569 (the α^P2 complex), EMD-30570 (the α^P1 complex), and EMD-30571 (eIF2B apo). The atomic coordinates are available at PDB, entry PDB: 7D43 (the $\alpha^P\gamma$ complex), PDB: 7D44 (the α^P2 complex), PDB: 7D45 (the α^P1 complex), and PDB: 7D46 (eIF2B apo).

EXPERIMENTAL MODEL AND SUBJECT DETAILS

T7 Express competent *E. coli* strain

T7 Express Competent *E. coli* strain (New England Biolabs, C2566I) was used for the expression of the human eIF2B$\beta\delta\gamma\varepsilon$ tetramer and eIF2Bα_2 dimer. Cultures were grown in Luria-Bertani broth supplemented with glucose in the orbital incubator shaker at 37°C and speed of 100 r.p.m, and induced by IPTG at 18°C. The full human eIF2B decamer was reconstituted by mixing the separately-purified eIF2B$\beta\delta\gamma\varepsilon$ tetramer and eIF2Bα_2 dimer.

FreeStyle 293-F cells

FreeStyle 293-F cells (embryonic kidney epithelial-derived, female, Thermo Fisher Scientific, R79007) were cultured in FreeStyle293 Expression Medium (Thermo Fisher Scientific, 12338) at 37°C in 8% CO_2 atmosphere, and were used for the overexpression of the human eIF2 trimer. The transfections of the plasmids were performed using polyethylenimine (Polyscience, 24765-1).

T7 Express lysY/Iq competent *E. coli* strain

T7 Express lysY/Iq competent *E. coli* strain (New England Biolabs, C3013) was used for expression of the eIF2α-NTD (biotinylated UK2733, or not UK2731). Cultures were grown in Luria-Bertani broth in the orbital incubator shaker at 37°C and speed of 200 rpm

CHO-K1-derived adherent cell lines

Chinese hamster ovarian epithelial cells (female) were maintained in Nutrient Mixture F12 (N4888, Sigma), 10% Fetal Calf serum (FetalClone II, Thermo), 2 mM L- glutamine (G7513, Sigma Aldrich), and 1 x Penicillin/Streptomycin (P0781, Sigma) at 37°C with 5% CO_2. These cells were used in the experiments described in Figures 1C, 1D, and 6. The generated cell lines have not been authenticated.

HeLa-derived adherent cell lines

Human cervical epithelial cells (female) were maintained in DMEM (D6546, Sigma Aldrich) supplemented with 2 mM L-glutamine (G7513, Sigma Aldrich), 1 x Penicillin/ Streptomycin (P0781, Sigma), 1 x non-essential amino acids solution (M7145, Sigma), and 55 μM β-mercaptoethanol at 37°C with 5% CO_2. These cells were used in the experiments described in Figure 6. These cells were used in the experiments described in Figure 6B. The generated cell lines have not been authenticated.

All the cell lines generated in this study are described in Table S1 and Key Resources Table.

METHOD DETAILS

Protein preparation

Human eIF2B, wild-type, or ISR-defective δ^{E310K} or δ^{L314Q} mutant versions, were purified from a bacterial expression system, whereas human eIF2, wild-type or non-phosphorylatable α^{S51A} mutant, were purified from transfected FreeStyle 293-F cells as previously described (Kashiwagi et al., 2019). As the α and γ subunits of eIF2 in this study have C-terminal PA and FLAG-His_8 tags, respectively, human eIF2 proteins were purified by a His-Accept column (Nacalai tesque), Anti PA tag Antibody Beads (Fujifilm Wako), and a HiTrap desalting column (GE Healthcare), and were dissolved in 20 mM HEPES-KOH buffer pH 7.5 containing 200 mM KCl, 1 mM $MgCl_2$, 1 mM DTT, and 10%($^V/_V$) glycerol. The N-terminally Sumo3 tagged and C-terminally-AviTagged N-terminal domain of human eIF2α (residues 1-187), were purified from bacteria (where the latter was biotinylated by the endogenous BirA) by Ni-NTA affinity chromatography, followed by cleavage of the Sumo3 tag with Senp2.

Purified human eIF2 or the biotinylated N-terminal domain of human eIF2α were phosphorylated *in vitro* in kinase buffer (20 mM HEPES-KOH pH 7.4, 150 mM KCl, 1 mM TCEP, 2 mM $MgCl_2$, 1 mM ATP) using bacterial-expressed PERK kinase domain (immobilised on glutathione Sepharose beads and removed from the reaction at conclusion by phase separation). Stoichiometric phosphorylation of the eIF2α subunit was confirmed on a Coomassie-stained PhosTag gel (see Figure S3A).

Guanine nucleotide exchange activity

eIF2B guanine nucleotide exchange activity was measured as described previously (Sekine et al., 2015), with minor modifications. Briefly, purified decameric eIF2B (final concentration 2.5 - 40 nM), phosphorylated or unphosphorylated eIF2 (final concentration 0 - 1 μM), ISRIB (a gift of Peter Fischer, U, Nottingham) dissolved in DMSO (final concentration 0.25 - 1 μM) or DMSO carrier control (final concentration < 5%$^V/_V$) were pre-assembled in assay buffer (20 mM HEPES-KOH pH 7.4, 150 mM KCl, 2 mM $MgCl_2$, 1 mM TCEP, 0.05 mg/mL bovine serum albumin, 0.01% Triton X-100, 1.5 mM GDP) and allowed to equilibrate for 10 minutes at room temperature in a low volume, "U" bottom, black 394 well plate (Corning, Cat #3667). At t = 0 purified eIF2(α^{S51A}), preloaded with BODIPY-FL-GDP (Invitrogen, G22360) (as previously described) (Sekine et al., 2015) was introduced at a final concentration of 125 nM and the fluorescence signal read kinetically in a Tecan F500 plate reader (Excitation wavelength: 485 nm, bandwidth 20 nm, Emission wavelength: 535 nm, bandwidth 25 nm). Where indicated, the data were fitted to a single-phase exponential decay function using GraphPad Prism V8: [Y = (Y0 - Plateau)*exp(-K*X) + Plateau], where Y0 is the Y value when X (time) is zero, Plateau is the Y value at infinite times, K is the rate constant expressed in reciprocal of the x axis time units.

Cryo-EM analysis

Cryo-EM sample preparation and data collection were performed as previously described (Kashiwagi et al., 2019), but the ratio of eIF2B and eIF2(αP) was changed to 1:4, and they were diluted to 60 nM and 240 nM, respectively. The total number of collected images was 7,729.

The movie frames were aligned with MotionCor2 (Zheng et al., 2017) and the CTF parameters were estimated with Gctf (Zhang, 2016) in RELION-3.0 (Zivanov et al., 2018). To make the templates for automated particle picking with Gautomatch (https://www.mrc-lmb.cam.ac.uk/kzhang/), about 20,000 particles were semi-automatically picked with EMAN2 (Tang et al., 2007), and 2D averages were generated in RELION. Automatically picked 1,889,101 particles were extracted with rescaling to 2.94 Å/pix and 2D & 3D classification in RELION was performed. A low-pass filtered (40 Å) map calculated from the crystal structure of *Schizosaccharomyces pombe* eIF2B (PDB: 5B04) (Kashiwagi et al., 2016) was used as a reference map in 3D classification. After 3D classification steps, 365,487 particles in good classes were re-extracted without rescaling (1.47 Å/pix), and 3D refinement, Bayesian polishing, and CTF refinement were performed. These refined particles were applied to 3D classification again, and separated into classes in which one molecule of eIF2α (the α^P1 complex, 208,728 particles, 3.8 Å), two molecules of eIF2α (the α^P2 complex, 80,921 particles, 4.0 Å), or eIF2αγ at one side and eIF2α at the other side are resolved (the $\alpha^P\gamma$ complex, 66,721 particles, 4.3 Å), respectively. In addition, the previous dataset for the eIF2B·eIF2(αP) complex (PDB: 6K72) (Kashiwagi et al., 2019) was also re-analyzed. The 3D class not containing eIF2(αP) was selected re-extracted and refined as above (330,601 particles, 4.0 Å).

As a model, the cryo-EM structure of human eIF2B in complex with P-eIF2α at 3.0-Å resolution (PDB: 6O9Z) (Kenner et al., 2019) was used for the most part of eIF2B and the N-terminal domain of P-eIF2α. For the rest, the cryo-EM structure of human eIF2B in complex with eIF2(αP) at 4.6-Å resolution (PDB: 6K72) (Kashiwagi et al., 2019) was used. These structures were manually fitted into the maps. Map sharpening and model refinement were performed in PHENIX (Adams et al., 2010), and the models were further refined manually with Coot (Emsley et al., 2010). The refinement statistics of these structures are shown in Table 1.

ISRIB binding to eIF2B

FAM-conjugated ISRIB (at 2.5 - 5 nM, final) (Zyryanova et al., 2018) was combined with purified eIF2B (6 - 150 nM) in presence or absence of phosphorylated or unphosphorylated eIF2 (final concentration 0 - 2.5 μM), the N-terminal domain of phosphorylated eIF2α (final concentration 0 - 40 μM) or unlabelled ISRIB (0.5 - 1 μM) in assay buffer above, and allowed to equilibrate for 30 minutes at room temperature in a low volume, "U" bottom, black 394 well plate (Corning, Cat #3667). In Figure S4B eIF2B was titrated into a buffer containing 2.5 nM FAM-ISRIB ± 15 μM eIF2α-NTD (UK2733), or ± 1 μM eIF2(α^{S51A}). The fluorescence polarization signal was read on a CLARIOstar microplate reader (BMG Labtech) with filter settings of 482 nm (excitation) and 530 nm (emission). The data was fitted and $K_{1/2max}$ was extracted using one site – total binding function: [Y = Bmax*X/(Kd+X) + NS*X + Background], where Bmax is the maximum specific binding, Kd is the equilibrium dissociation constant reporting on the radioligand concentration needed to achieve a half-maximum binding at equilibrium in the same units as X, NS is the slope of nonspecific binding in Y units divided by X units, Background is the amount of nonspecific binding with no added radioligand; inhibition data was fitted using the log(inhibitor) versus response – variable slope (four parameters) function: [Y = Bottom + (Top-Bottom)/(1+10^((LogIC_{50}-X)*HillSlope)], where IC_{50} is the concentration of agonist that gives a response half way between Bottom and Top, HillSlope describes the steepness of the family of curves, Top and Bottom are plateaus in the units of the y axis - on GraphPad Prism V8.

Where indicated (Figure 4A) at t = 0 unlabelled ISRIB (1 μM final) or equal volume of DMSO carrier were introduced into samples containing pre-equilibrated FAM-ISRIB (a gift of Peter Fischer, U. Nottingham) and eIF2B (60 nM) and the fluorescence polarization signal was read kinetically. The data were fitted to a single-phase exponential decay function using GraphPad Prism V8: [Y = (Y0 - Plateau)*exp(-K*X) + Plateau], where Y0 is the Y value when X (time) is zero, Plateau is the Y value at infinite times, K is the rate constant expressed in reciprocal of the x axis time units.

Where indicated (Figures 4B and S3A) at t = 0 PERK kinase (1 to 100 nM final concentration, of bacterially-expressed GST-PERK) was introduced into samples containing pre-equilibrated FAM-ISRIB (2.5 - 5 nM), eIF2B (60 - 83 nM), wild-type eIF2 or non-phosphorylatable eIF2(α^{S51A}) (300 - 600 nM) in assay buffer supplemented with 1 mM ATP and the change in fluorescence polarization was read kinetically.

Where indicated (Figure S3B), at t = 0 bacterially expressed lambda phosphatase (160 nM) or a pre-assembled complex of the trimeric eIF2(αP)-directed holophosphatase comprised of G-actin/PP1A catalytic subunit/PPP1R15A regulatory subunit (as described in Crespillo-Casado et al. [2018], final concentration, 100 nM G-actin, 100 nM PPP1R15A, 10 nM PP1A) was introduced in samples with pre-equilibrated FAM-ISRIB (2.5 nM), eIF2B (60 nM) and eIF2(αP) (300 nM) and the change in fluorescence polarization was read kinetically.

eIF2B binding to phosphorylated eIF2α

BLI experiments were conducted at 30°C on the ForteBio Octet RED96 System, at an orbital shake speed of 600 rpm, using Streptavidin (SA)-coated biosensors (Pall ForteBio) in 20 mM HEPES pH 7.4, 150 mM KCl, 2 mM $MgCl_2$, 1 mM TCEP, 0.05 mg/mL bovine serum albumin and 0.01% Triton X-100. Biotinylated ligand [C-terminally-AviTag-His_6 tagged N-terminal domain of human eIF2α (residues 1-187 at a concentration of 150 nM)] was loaded to a binding signal of 1-2 nm, followed by baseline equilibration in buffer. Association reactions with analyte (wild-type or mutant eIF2B decamers or eIF2Bβδγε tetramers) prepared in the aforementioned buffer, or dissociation reactions in buffer, with ISRIB or an equal volume of DMSO were conducted with a reaction volume of 200 μL in 96-well microplates (greiner bio-one). In Figure 5C association reactions were conducted without ISRIB and the dissociation reactions (shown) were conducted with the indicated concentration of ISRIB, and equal final volumes of DMSO. In Figure S4A dissociation was measured in the buffer containing respective amounts of ISRIB or DMSO ± 4.5 μM eIF2α-NTD (UK2731).

Data were analyzed using Prism GraphPad V8, as indicated in the figure legends.

Two-phase association: [SpanFast = (Plateau-Y0)*PercentFast*.01]; [SpanSlow = (Plateau-Y0)*(100-PercentFast)*.01]; [Y = Y0+SpanFast*(1-exp(-KFast*X)) + SpanSlow*(1-exp(-KSlow*X))], where Y0 is the Y value when X (time) is zero, Plateau is the Y value at infinite times, Kfast and Kslow are the two rate constant expressed in reciprocal of the x axis time units, PercentFast is the fraction of the span (from Y0 to Plateau) accounted for by the faster of the two components.

Two-phase decay: [SpanFast = (Y0-Plateau)*PercentFast*.01]; [SpanSlow = (Y0-Plateau)*(100-PercentFast)*.01]; [Y = Plateau+SpanFast*exp(-KFast*X)+SpanSlow*exp(-KSlow*X)], where parameters are as above.

One-site specific binding Hill slope = 1: [Y = Bmax*X/(Kd + X)], where Bmax is the maximum specific binding in the same units as Y, Kd is the equilibrium dissociation constant in the same units as X.

[Agonist] versus response Hill slope = 1: [Y = Bottom + X*(Top-Bottom)/(EC_{50} + X)], where EC_{50} is the concentration of agonist that gives a response half way between Bottom and Top, Top and Bottom are plateaus in the units of the y axis.

Measurement of the ISR in cultured cells

Generation of CHO-S21 and CHO-S21 *Eif2S1*^{S51A} cells containing a stably integrated ISR (CHOP::GFP) and UPR (Xbp1::Turquoise) responsive reporter was described previously (Sekine et al., 2016). Inhibition of histidyl-tRNA synthetase by histidinol in the parental CHO-S21 cells, but not in the *Eif2S1*^{S51A} mutant, activates the eIF2α kinase GCN2 that phosphorylates eIF2. eIF2(αP) inhibits its GEF eIF2B, initiating the ISR, and culminating in CHOP::GFP activation, which was detected by flow cytometry. In wild-type histdinol-treated cells the presence of ISRIB attenuates the response of the CHOP::GFP reporter, however, in eIF2(αS51A) mutant cells this effect can no longer be observed due to inability of histidinol to trigger the ISR response in those cells (also known as gcn-phenotype).

To observe the drugs effect in any of CHO cell lines, cells were split and seeded at confluency of 2 - 4 × 10^4 cells/well on a 12-well plate. Two days later the medium was refreshed and cells were either treated with 0.5 mM L-histidinol (228830010, Acros Organics), or 200 nM ISRIB, or both for 18 - 24 hours. Immediately before flow cytometry analysis, cells were washed with ice-cold PBS, and collected in ice-cold PBS containing 4 mM EDTA pH 8.0. Fluorescent signal from single cells (10,000/ sample) was measured on LSRFortessa cell analyzer.

The populations of cells were further analyzed on FlowJo software where the median for each Gaussian distribution was defined. For the samples containing bimodal distribution two medians were defined. To assess the ISR folds increase in each transfected sample the median of the "ISR-on" CHOP::GFP signal (right distribution) was divided by the median of the "ISR-off" CHOP::GFP signal (left distribution). In the case of a unimodal distribution the median of a given population was divided on itself. The means of three repeats with standard deviations and P values were obtained using Prism software.

eIF2B and eIF2 subunits depletion

CHO-S21 *Eif2S1*^{S51A} cells were split and seeded at density of 5 × 10^4 cells/well on a 12-well plate. The next day cells of about 20%–30% confluence were pre-treated for 60 minutes with either 1 μM of ISRIB in DMSO or the equivalent amount of 100% DMSO and then transfected with 1 μg of CRISPR/ Cas9 plasmid either without (control) of with sgRNA (see Table S2 for plasmids and Table S3 for primers) using Lipofectamine LTX with Plus Reagent (A12621, Thermofisher) according to the manufacturer's protocol. Medium supplemented with either 1 μM ISRIB or the equivalent amount of 100% DMSO was refreshed every 24 hours thereafter. On the day of harvest (48, 72 and 96 hours post transfection) cells were washed twice with ice-cold PBS, harvested in 0.5 mL of ice-cold PBS supplemented with 4 mM EDTA and immediately analyzed on LSRFortessa cell analyzer. CHOP::GFP (excitation 488 nm/ emission 530 ± 30 nm) fluorescence signal from single cells (20,000/ sample) was measured. The populations of cells were further analyzed on FlowJo software as described above.

Introduction of ISR resistant mutations into cultured cells

Assessing the importance of counterparts to *S. cerevisiae* eIF2Bδ residues GCD2^{E377} and GCD2^{L381} (known ΔISR/ΔGCN yeast mutants, Pavitt et al., 1997, E312 and L316 in the hamster genome, and E310 and L314 in the human) to the ability of eIF2B to respond to eIF2(αP) and initiate an ISR *in vivo*, was carried out by targeting the *Eif2b4* locus of CHOP::GFP carrying CHO-S21 cells (described above) with a CRISPR/Cas9 guide (GAAGATTGTGCTTGCAGCTC**AGG**, PAM sequence in bold) and providing an ssODN repair template randomized at codon L316 and either carrying the wild-type sequence at E312 (oligo #2213, eIF2B4_ ssODN_L316X) or an additional E312K mutation (oligo #2214, eIF2B4_ ssODN_E312K_L316X) (Table S3) . The transduced cells were selected for ISR resistance based on defective CHOP::GFP induction in response to histidinol. Single clones were sequenced, two of which 12H6 (genotype *Eif2b4*^{L316N}) and 22H2 (genotype *Eif2b4*$^{E312K; L316V}$) were selected for further study (Figure 1C; Table S1).

The effect of the mutations on translational control in response to stress was assessed by measuring the incorporation of puromycin into newly synthesized proteins by immunoblotting lysates of untreated and thapsigargin (Sigma, T9033) (200 nM, 45')-treated cells that had been exposed to 10 μg/mL puromycin (Sigma, P8833) 10 minutes before lysis. Immunoblot detection was conducted using primary antibodies for puromycinylated protein (Schmidt et al., 2009), phospho-eIF2α-Ser51 (Epitomics), or total eIF2α (Scorsone et al., 1987), and IR800 or IR680 conjugated secondary antisera followed by scanning on a Li-Cor Odyssey scanner. The extent of the ISR defect was benchmarked against CHO-S21 cells with an *Eif2S1*^{S51A} knock-in mutation (Sekine et al., 2016). Blot signals were quantified using Fiji (ImageJ 1.53c, National Institute of Health, USA) (Schindelin et al., 2012).

Glycerol gradient fractionation of cell lysates

CHO-S7 [with a 3XFLAG tag knocked into their *Eif2b3* locus (*Eif2b3*$^{3xFLAG\ in/+}$)] (Sekine et al., 2016) and HeLa [with a 3XFLAG tag knocked into their *EIF2B2* locus (*EIF2B2*$^{3xFLAG\ in/in}$)] (Sekine et al., 2015) cells (9 × 10^7 cells/ sample) were harvested, lysed in 250-500 μL of lysis buffer (50 mM HEPES-KOH pH 7.5, 150 mM NaCl, 1% (v/v) Triton, 5% (v/v) Glycerol, 1mM DTT, 2 mM PMSF, 8 μg/ml aprotinin, 4 μg/mL pepstatin) either with 250 nM ISRIB (in DMSO) or equivalent amount of 100% DMSO, and cleared supernatant was applied on 5 mL of 10 - 40% (v/v) glycerol gradient prepared in lysis buffer (without triton) with respective amounts of glycerol using SG15 Hoefer Gradient Maker and centrifuged using SW50 (Beckman Coulter) rotor at 45,000 rpm for either 13 hours or for 14 hours 48 minutes at 4°C. After the centrifugation gradients were manually fractionated into 16 fractions of 325 μL, and 30 μL of each fraction was taken for western blot analysis. Fractions were run on 10% SDS-PAGE gel, transferred onto PVDF membrane, incubated for 2 hours at RT with primary monoclonal mouse anti-FLAG M2 antibody (F1804, Sigma Aldrich) to track migration of 3 x FLAG-tagged eIF2B complex, followed by incubation for 45 min at RT with secondary goat anti-mouse-HRP antibodies according to

the manufacturer's protocol. Membranes were developed with enhanced chemiluminescence kit following the manufacturer's procedure, and scanned on CheminDoc (Bio-Rad). Image analysis was done using ImageJ software.

QUANTIFICATION AND STATISTICAL ANALYSIS

For all the statistical and quantitative analysis we used the predetermined functions in Graphpad Prism V8. All the details on the model fitting equations and statistical tests with 'n' values are indicated in the relevant figure legends and method sections.

Molecular Cell, Volume *81*

Supplemental Information

ISRIB Blunts the Integrated Stress Response by Allosterically Antagonising the Inhibitory Effect of Phosphorylated eIF2 on eIF2B

Alisa F. Zyryanova, Kazuhiro Kashiwagi, Claudia Rato, Heather P. Harding, Ana Crespillo-Casado, Luke A. Perera, Ayako Sakamoto, Madoka Nishimoto, Mayumi Yonemochi, Mikako Shirouzu, Takuhiro Ito, and David Ron

Supplemental materials

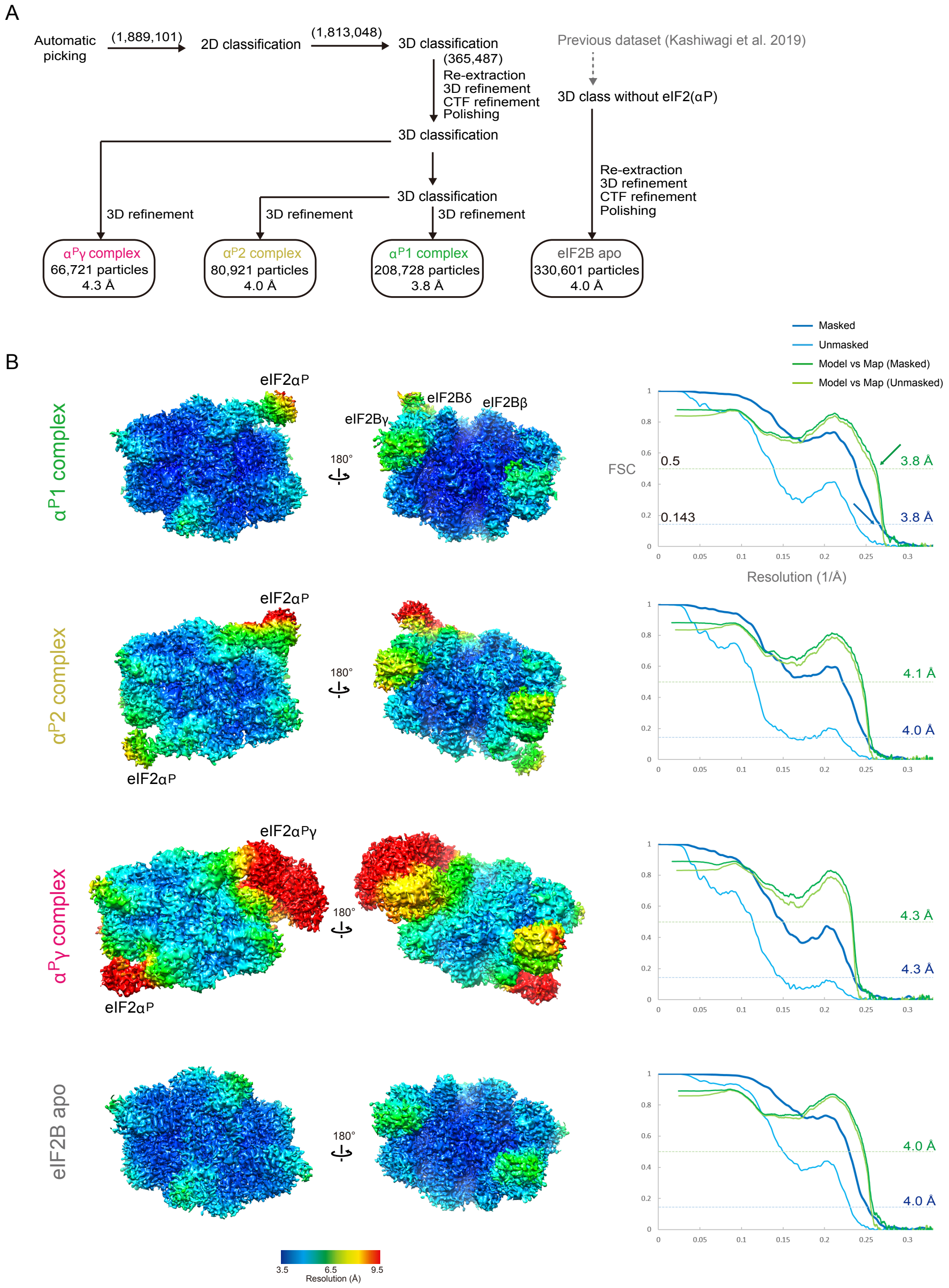

Figure S1. Cryo-EM data processing, related to Figure 2 and Table 1.

A) Workflow of image processing. Total particle numbers at each stage are show in parentheses.

B) Local resolution maps and Fourier shell correlation (FSC) curves of the cryo-EM maps. The FSC curves for masked (blue), unmasked (cyan) map, and the curves for model and map correlation (masked: green, unmasked: yellow green) are shown. The resolutions at which FSC for masked map drops below 0.143 and model map correlation drops below 0.5 are shown.

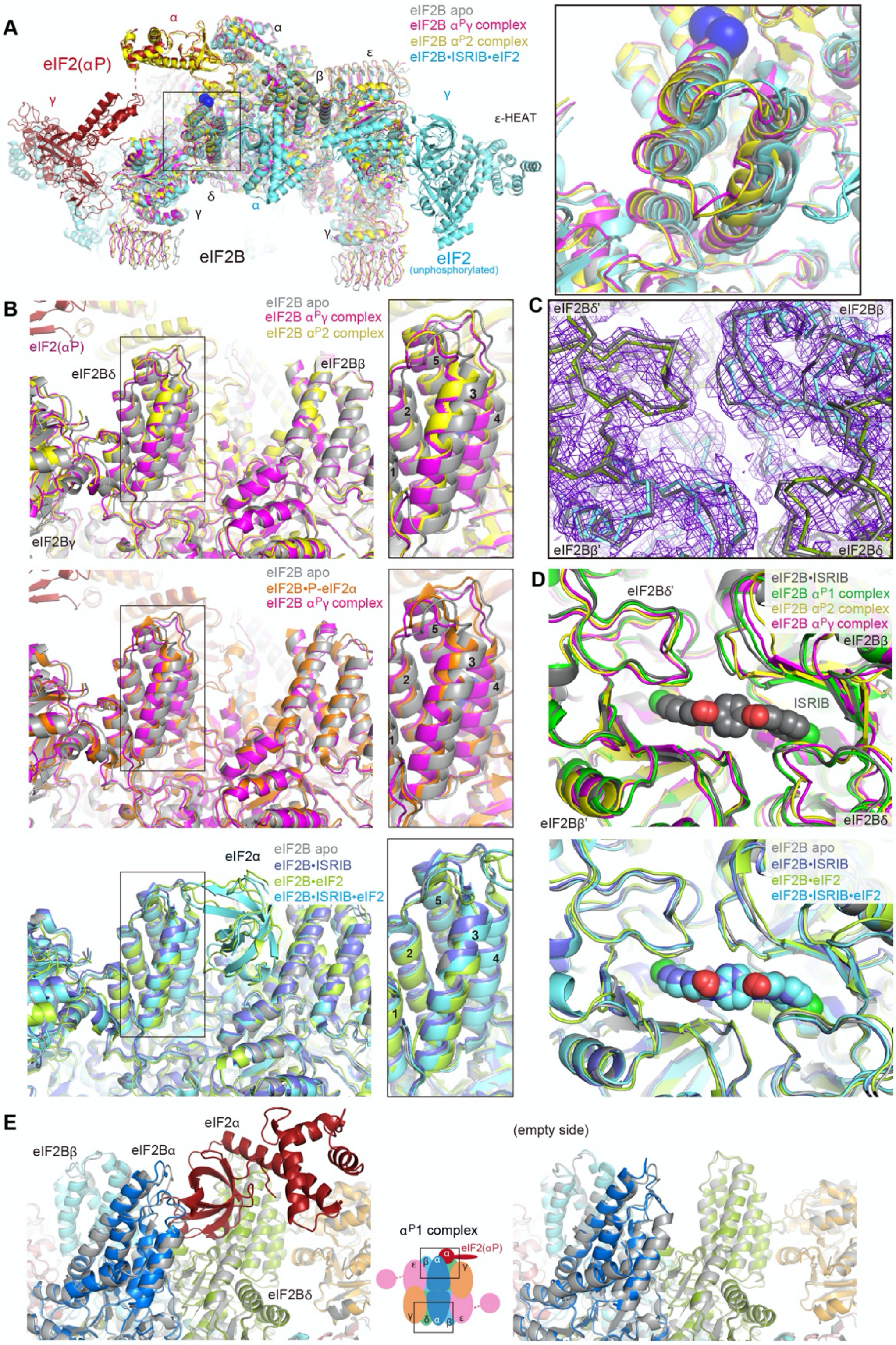

Figure S2. Changes to the catalytically productive interface and the ISRIB-binding pocket of eIF2B induced by eIF2(αP) binding, related to Figure 2.

A) Overlay of the eIF2B apo structure (grey), the $\alpha^P\gamma$ complex (eIF2B in magenta and eIF2(αP) in red), the α^P2 complex (all in yellow), and the eIF2B•ISRIB•eIF2 complex (all in cyan, PDB: 6O81) (same view as Figure 2A). Right panel: Close-up view of the displacement of the N-terminal helices of eIF2Bδ by the binding of unphosphorylated (cyan) or phosphorylated eIF2 (magenta). The blue spheres show the position of the C^α atoms of eIF2Bδ^{E310} and δ^{L314}.

B) Comparisons of the catalytically-productive pocket of eIF2B in various structures (same view as Figure 2B). Upper panels: comparison of apo eIF2B (grey) and eIF2B complexes with the eIF2(αP) trimer in which eIF2βγ is resolved ($\alpha^P\gamma$, magenta), or unresolved (α^P2, yellow). Middle panels: comparison of apo eIF2B (grey), the $\alpha^P\gamma$ complex (magenta) and the complex with the isolated phosphorylated eIF2α (P-eIF2α) (PDB: 6O9Z, orange). Lower panels: comparison of apo eF2B (grey), the eIF2B•ISRIB complex (PDB: 6CAJ, blue), the eIF2B•eIF2 complex (PDB: 6K71, lime), and the eIF2B•ISRIB•eIF2 complex (cyan). Right panels are close-up views of the helix δ-α3. Note that eIF2Bδ of the α^P2 complex shows a similar displacement of eIF2Bδ as the $\alpha^P\gamma$ complex structure (upper panels), while eIF2Bδ of the eIF2B•P-eIF2α complex resides in a position intermediate between apo eIF2B and the $\alpha^P\gamma$ complex (middle panels). In both the eIF2B•eIF2 complex and the eIF2B•ISRIB•eIF2 complex, eIF2Bδ closes around eIF2α, while there is little displacement of eIF2Bδ in the eIF2B•ISRIB complex (lower panels).

C) Close-up view of the ISRIB-binding pocket at the eIF2B's β-δ interface (same view as Figure 2C). The EM density map for the $\alpha^P\gamma$ complex is shown in purple.

D) Views of the ISRIB-binding pocket (same view as Figure 2C). Upper panel: the comparison of apo eIF2B (grey), the α^P1 complex (green), the α^P2 complex (yellow), and the $\alpha^P\gamma$ complex (magenta). Lower panel: comparison of apo eIF2B (grey), the eIF2B•ISRIB complex (blue), the eIF2B•eIF2 complex (lime), and the eIF2B•ISRIB•eIF2 complex (cyan). Note the similar displacement between the β-δ heterodimeric units in the $\alpha^P\gamma$ complex and the α^P2 complex, but not in other structures.

E) Comparison of the eIF2Bα_2 homodimeric unit of the α^P1 complex (color-coded as in the adjacent cartoon) relative to the eIF2B apo structure (grey). Accommodation of a single molecule of eIF2(αP) induces only minor displacement at tips of the eIF2Bα_2 homodimer.

Structures are aligned by the four C-terminal domains of the β- and δ-subunits of eIF2B for A), B), E), and by the C^α atoms surrounding (within 10 Å) the ISRIB molecule in the eIF2B•ISRIB structure for C), D).

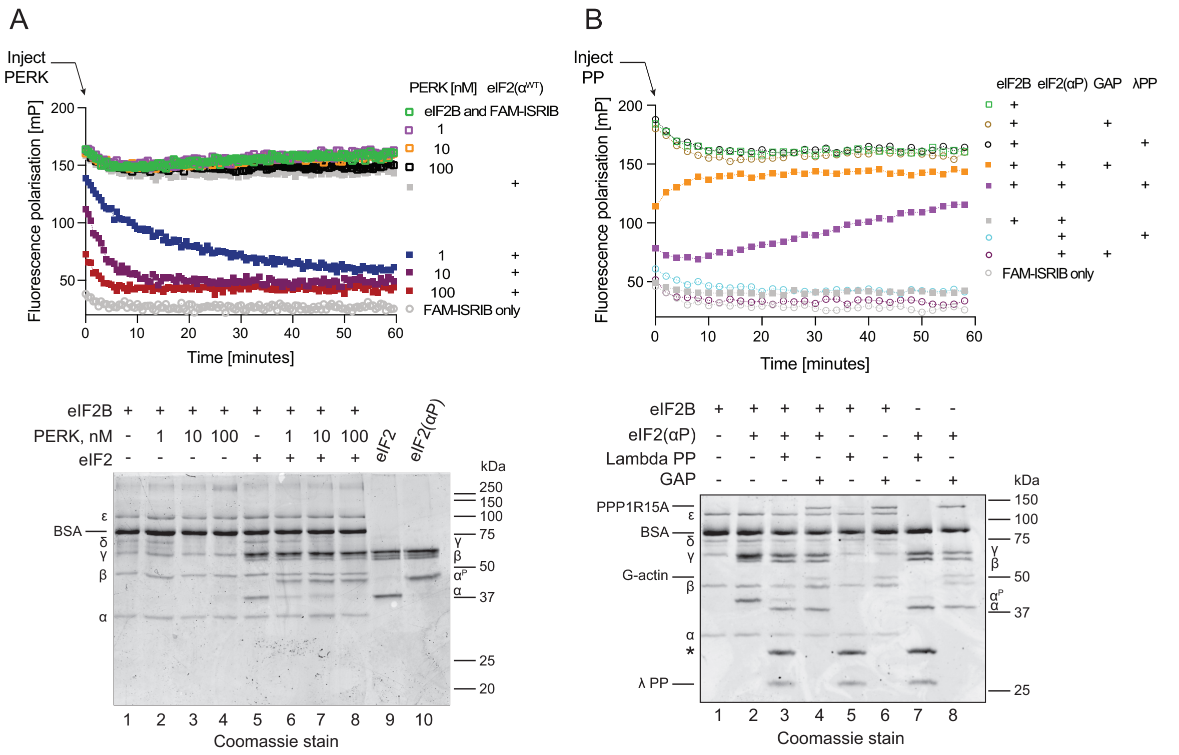

Figure S3. eIF2(αP)-mediated inhibition of FAM-ISRIB binding to eIF2B is captured kinetically and is reversible by dephosphorylation, related to Figure 4.

A) Upper left panel: plot of time-dependent change in fluorescence polarisation of FAM-ISRIB bound to wildtype eIF2B in presence or absence of unphosphorylated eIF2. Where indicated, at t = 0 the eIF2α kinase PERK was introduced at varying concentrations to promote a pool of eIF2(αP). Shown is a representative experiment (one of three).

Lower left panel: Coomassie-stained PhosTag SDS-PAGE of the samples analysed in the experiment above. Migration of the eIF2 subunits, including phosphorylated and unphosphorylated eIF2α, are indicated on the right. Pure samples of unphosphorylated and phosphorylated eIF2 are provided as references. The prominent band at ~70 kDa present in all lanes is bovine serum albumin (BSA), utilised as a stabiliser in all reactions (it obscures the GST-PERK signal, where applicable). Migration of eIF2B subunits is indicated on the left.

B) Upper right panel: Plot of time-dependent change in fluorescence polarisation of FAM-ISRIB bound to wildtype eIF2B in presence or absence of phosphorylated eIF2. Where indicated, at t = 0 a specific eIF2(αP)-directed holophosphatase consisting of G-actin/PP1A/PPP1R15A (GAP) or the non-specific lambda phosphatase (λP) was introduced to convert phosphorylated eIF2 to eIF2. Shown is a representative experiment (one of two).

Lower right panel: Coomassie-stained PhosTag SDS-PAGE gel of the samples analysed in the experiment above. The eIF2 subunit, including phosphorylated and unphosphorylated eIF2α, are indicated on the right, eIF2B subunits and species arising from the phosphatase-treated samples are indicated on the left (the catalytic subunit PP1A is not visible on this gel; the asterisk marks an unidentified contaminant of the λP samples).

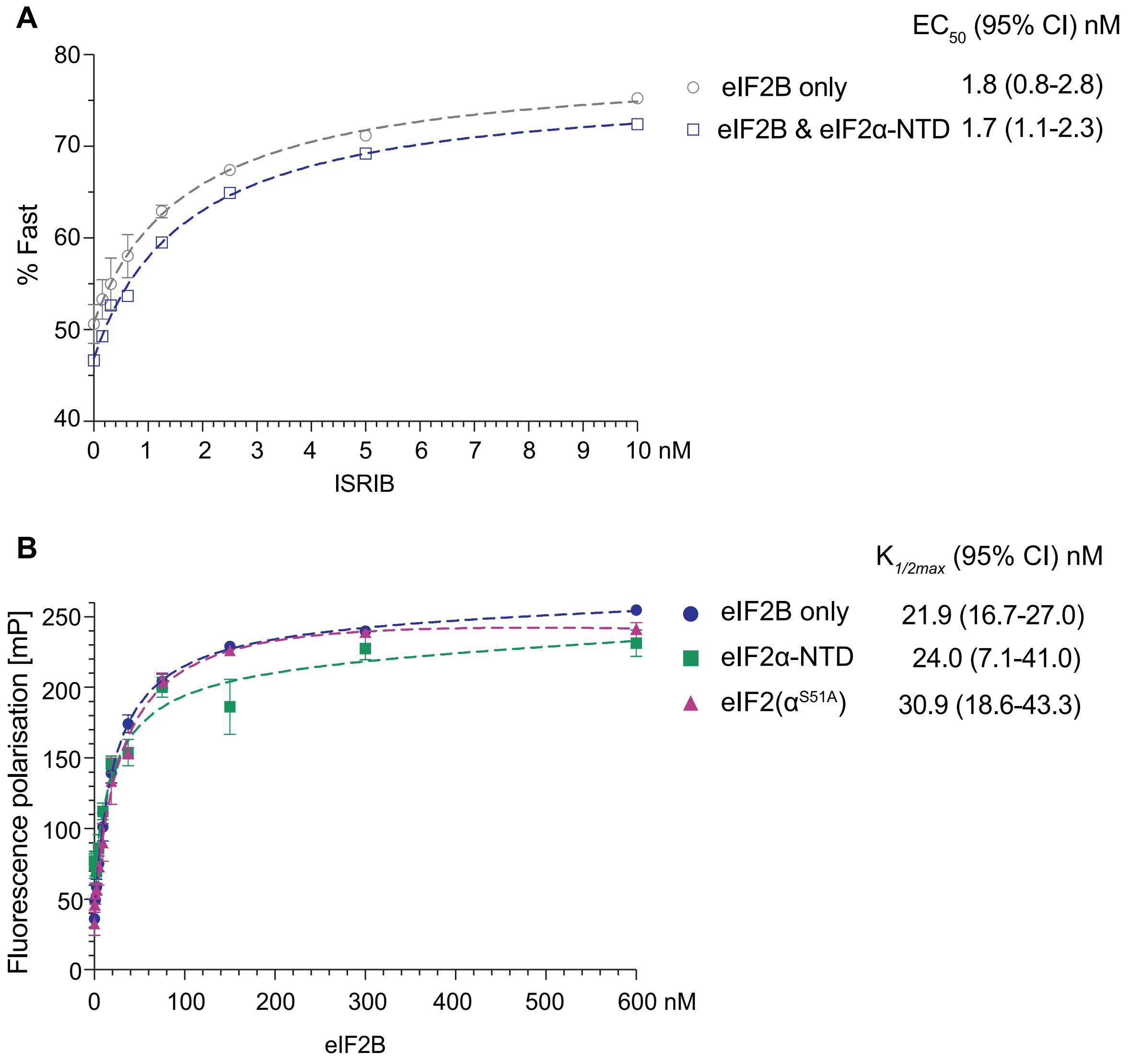

Supplementary Figure 4. Lack of cooperativity between unphosphorylated eIF2 and ISRIB in binding to eIF2B, related to Figure 5.

A) Unphosphorylated eIF2α-NTD does not affect the dissociation of eIF2B from P-eIF2α-NTD in the presence of ISRIB. As in Figure 5C (right plot): plot of the %Fast of the dissociation reactions as a function of ISRIB concentration, obtained through BLI experiment monitoring dissociation of eIF2B from immobilised P-eIF2α-NTD in the presence of indicated concentrations of ISRIB. The grey curve (eIF2B only) is redrawn from Figure 5C (right plot), the blue curve indicates the dissociation performed in the presence of unphosphorylated eIF2α-NTD. The data was fitted to an [Agonist] vs. response (Hill slope = 1) non-linear regression model (dotted line). EC_{50} with 95% CI is indicated.

B) Presence of unphosphorylated eIF2 does not affect binding of FAM-ISRIB to eIF2B. Plot of fluorescence polarisation signals (mean ± SD, n=3) arising from samples of FAM-conjugated ISRIB (2.5 nM) incubated with varying concentrations of wildtype eIF2B. Where indicated 15 µM eIF2α-NTD or 1 µM eIF2(α^{S51A}) was added. $K_{1/2max}$ with 95% CI is shown. The difference in $K_{1/2max}$ values in these experiments, compared with those shown in Figure 3A, likely reflect differences in eIF2B preparations.

Table S1. List of cell lines, related to Figure 1C & D, and Figure 6.

Gene	Exon	Cells	Clone name	Description	Mutagenized region (numbers indicate amino acid position at which mutagenesis occurred)
NA	NA	CHO-S21	NA	dual reporter [CHOP::GFP; Xbp1::Turquoise] parental cell line from Sekine et al. 2016	NA
Eif2S1	2	CHO S51A	NA	dual reporter ISR-insensitive (gcn-) eIF2α^{S51A} from Sekine et al. 2016	51_**A**RRRIRSI
Eif2b4	10	CHO-S21	12H6	eIF2Bδ(L316N), ISR-insensitive	316_**N**AAQAISRF
Eif2b4	10	CHO-S21	22H2	eIF2Bδ(E312K; L316V), ISR-insensitive	312_**K**KIV_316_**V**AAQA
Eif2b3	11	CHO-C30	S7	CHOP::GFP-reporter eIF2Bγ-3xFlag-tagged cells from Zyryanova et al. 2018	451_EFCRYPAQWRPLERADYKDHDGDYKDHDIDYKDDDDK*
EIF2B2	1	HeLa	2C2	3 X Flag-tagged eIF2Bβ cells from Sekine et al. 2015	2_PGSDYKDHDGDYKDHDIDYKDDDDK
FreeStyle 293-F cells	NA	HEK293	NA	Mammalian expression cell line	NA

Table S2. List of plasmids, related to Figures as indicated.

ID	Plasmid name	Description	Primers used to generate plasmid	Figures
UK2320	CHO_EIF2B4_EXON10_g3_pSpCas9(BB)-2A-Puro	CRISPR/ Cas9 with puromycin selection targeting hamster *Eif2b4* (eIF2B delta) gene	Oligo 2209 & 2210	1C
UK2733	heIF2a_2-187_WT_AviTag_H6_pET-30a(+)	wildtype NTD human eIF2alpha_1-187 with AviTag and 6x histidines in bacterial expression vector	NA	3B
UK1610	pSpCas9(BB)-2A-mCherry_V2	CRISPR/ Cas9 empty vector with mCherry selection	NA	5A
UK2536	cgeIF2B2_g2_pSpCas9(BB)-2A-mCherry	CRISPR/ Cas9 with mCherry selection targeting hamster *Eif2b2* (eIF2B beta) gene (guide 1)	Oligo 2520 & 2521	5A
UK2537	cgeIF2B2_g3_pSpCas9(BB)-2A-mCherry	CRISPR/ Cas9 with mCherry selection targeting hamster *Eif2b2* (eIF2B beta) gene (guide 2)	Oligo 2522 & 2523	5A
UK2538	cgeIF2B4_g1_pSpCas9(BB)-2A-mCherry	CRISPR/ Cas9 with mCherry selection targeting hamster *Eif2b4* (eIF2B delta) gene (guide 1)	Oligo 2524 & 2525	5A
UK2539	cgeIF2B4_g3_pSpCas9(BB)-2A-mCherry	CRISPR/ Cas9 with mCherry selection targeting hamster *Eif2b4* (eIF2B delta) gene (guide 2)	Oligo 2526 & 2527	5A
UK2547	cgeIF2B5_g1_pSpCas9(BB)-2A-mCherry	CRISPR/ Cas9 with mCherry selection targeting hamster *Eif2b5* (eIF2B epsilon) gene	Oligo 2543 & 2544	5A
UK1367	pSpCas9(BB)-2A-Puro	CRISPR/ Cas9 empty vector with puromycin selection	NA	5C
UK1505	CHO_Eif2s1_guideA_pSpCas9(BB)-2A-Puro	CRISPR/ Cas9 with puromycin selection targeting hamster *Eif2s1* (eIF2 alpha) gene (guide A)	Oligo 1015 & 1018	5C
UK1506	CHO_Eif2s1_guideB_pSpCas9(BB)-2A-Puro	CRISPR/ Cas9 with puromycin selection targeting hamster *Eif2s1* (eIF2 alpha) gene (guide B)	Oligo 1016 & 1019	5C
UK2731	heIF2a_2-187_pSUMO3	encodes H6-SUMO3-SER_hueIF2a_2-187	NA	S4A
NA	*pET28-3C-2B1*	For bacterial expression of eIF2B alpha from Kashiwagi et al. 2019	NA	1A&B, 2-5, S1, S2
NA	*pETDuet-2B4-2B2*	For bacterial expression of eIF2B delta and beta from Kashiwagi et al. 2019	NA	1A&B, 2-5, S1-2
NA	*pCOLADuet-2B5-2B3*	For bacterial expression of eIF2B epsilon and gamma from Kashiwagi et al. 2019	NA	1A&B, 2-5, S1-2
NA	*pETDuet-2B4-2B2_dE310K*	For bacterial expression of eIF2B delta-E310K and beta	NA	1B, 3A&C, 4B
NA	*pETDuet-2B4-2B2_dL314Q*	For bacterial expression of eIF2B delta-L314Q and beta	NA	1B, 3A&C, 4B
NA	*pEBMulti-Neo-human-eIF2alpha*	For mammalian expression of eIF2 alpha from Kashiwagi et al. 2019	NA	2, S1-2
NA	*pEBMulti-Neo-human-eIF2alpha-PA*	For mammalian expression of eIF2 alpha	NA	3B&C, 4B, S3
NA	*pEBMulti-Neo-human-eIF2alpha-S52A-PA*	For mammalian expression of eIF2 alpha-S51A	NA	1A&B, 4B, S4B
NA	*pEBMulti-Neo-human-eIF2beta*	For mammalian expression of eIF2 beta from Kashiwagi et al. 2019	NA	1A&B, 2, 3B&C, 4B, S1-3, S4B
NA	*pEBMulti-Neo-human-eIF2gamma-FlagHis8*	For mammalian expression of eIF2 gamma from Kashiwagi et al. 2019	NA	1A&B, 2, 3B&C, 4B, S1-3, S4B

Table S3. List of primers, related to Figures as indicated.

ID	Oligo name	Sequence	Description	Figures
Oligo 2209	CHO_EIF2B4_EXON10_g3_1s	CACCGAAGATTGTGCTTGCAGCTC	sense primer to create UK2320 with sgRNA targeting hamster *Eif2b4* gene	1C
Oligo 2210	CHO_EIF2B4_EXON10_g3_2AS	AAACGAGCTGCAAGCACAATCTTC	anti-sense to create UK2320 with sgRNA targeting hamster *Eif2b4* gene	1C
Oligo 2213	CHO_eIF2B4_Exon10_ssODN_L310X	GGTTTTTCAGTCAGGTATTCACCAT<br>ACCATCCATATACCAGGATCACGTC<br>CCCGTCACTGATCTTCTTAGAGGCA<br>AACCGTGAAATTGCTTGAGCTGC**NN**<br>**N**CACAATCTTCTCTTGTACATACCGA<br>TCAATGGCTTCTCTAAGTTCTGACTT<br>TGCCTAAATGTTGAGAGAACAGTGA<br>TATAATTCACCC	single strand ODN repair template introducing eIF2Bδ(L316N), ISR-insensitive phenotype	1C
Oligo 2214	CHO_eIF2B4_Exon10_ssODN_E306K_L310X	GGTTTTTCAGTCAGGTATTCACCAT<br>ACCATCCATATACCAGGATCACGTC<br>CCCGTCACTGATCTTCTTAGAGGCA<br>AACCGTGAAATTGCTTGAGCTGC**NN**<br>**N**CACAATCTTCTTTTGTACATACCGA<br>TCAATGGCTTCTCTAAGTTCTGACTT<br>TGCCTAAATGTTGAGAGAACAGTGA<br>TATAATTCACCC	single strand ODN repair template introducing eIF2Bδ(E312K; L316V), ISR-insensitive phenotype	1C
Oligo 2520	cgeIF2B2_g2_S	CACCGCACACTCGGCAACAATGACA	sense primer to create UK2536 with sgRNA targeting hamster *Eif2b2* gene (guide 1)	5A
Oligo 2521	cgeIF2B2_g2_AS	AAACTGTCATTGTTGCCGAGTGTGC	anti-sense to create UK2536 with sgRNA targeting hamster *Eif2b2* gene (guide 1)	5A
Oligo 2522	cgeIF2B2_g3_S	CACCGATGGGTGCACACACGATGAG	sense primer to create UK2537 with sgRNA targeting hamster *Eif2b2* gene (guide 2)	5A
Oligo 2523	cgeIF2B2_g3_AS	AAACCTCATCGTGTGTGCACCCATC	anti-sense to create UK2537 with sgRNA targeting hamster *Eif2b2* gene (guide 2)	5A
Oligo 2524	cgeIF2B4_g1_S	CACCGATTATGCGCTCGAGCTACGA	sense primer to create UK2538 with sgRNA targeting hamster *Eif2b4* gene (guide 1)	5A
Oligo 2525	cgeIF2B4_g1_AS	AAACTCGTAGCTCGAGCGCATAATC	anti-sense to create UK2538 with sgRNA targeting hamster *Eif2b4* gene (guide 1)	5A

Oligo 2526	cgeIF2B4_g3_S	CACCGGAACCGCCTGCCCTCGACCC	sense primer to create UK2538 with sgRNA targeting hamster *Eif2b4* gene (guide 2)	5A
Oligo 2527	cgeIF2B4_g3_AS	AAACGGGTCGAGGGCAGGCGGTTCC	anti-sense to create UK2538 with sgRNA targeting hamster *Eif2b4* gene (guide 2)	5A
Oligo 2543	cgeIF2B5_g1_S	CACCGGAACAAAATCATCTCGAGTT	sense primer to create UK2547 with sgRNA targeting hamster *Eif2b5* gene	5A
Oligo 2544	cgeIF2B5_g1_AS	AAACAACTCGAGATGATTTTGTTCC	anti-sense to create UK2547 with sgRNA targeting hamster *Eif2b5* gene	5A
Oligo 1015	CHO_eif2s1_CrispyA_1s	CACCGTATTCCAACAAGCTAACAT	sense primer to create UK1505 with sgRNA targeting hamster *Eif2s1* gene (guide A)	5C
Oligo 1018	CHO_eif2s1_CrispyA_2AS	AAACATGTTAGCTTGTTGGAATAC	anti-sense primer to create UK1505 with sgRNA targeting hamster *Eif2s1* gene (guide A)	5C
Oligo 1016	CHO_eif2s1_CrispyB_1s	CACCGGGAGCCTATGTTAGCTTGT	sense primer to create UK1506 with sgRNA targeting hamster *Eif2s1* gene (guide B)	5C
Oligo 1019	CHO_eif2s1_CrispyB_2AS	AAACACAAGCTAACATAGGCTCCC	anti-sense primer to create UK1506 with sgRNA targeting hamster *Eif2s1* gene (guide B)	5C
